# Cross-tissue dual-omics analysis reveals molecular programs linked to myopia susceptibility and progression

**DOI:** 10.26508/lsa.202503595

**Published:** 2026-06-29

**Authors:** Liqin Jiang, Lijin Wang, Lei Zhou, Fangquan Wu, Johnny Kwok Kit Ma, Willie SS Yu, James HZ Koh, Audrey WL Chia, Seang-Mei Saw, Sujoy Ghosh, Veluchamy A Barathi

**Affiliations:** 1 Singapore Eye Research Institute , Singapore National Eye Centre, Singapore, Singapore; 2 Academic Clinical Program, Duke-NUS Medical School, Singapore, Singapore; 3 Centre for Computational Biology, Duke-NUS Medical School, Singapore, Singapore; 4 Department of Biochemistry and Molecular Biology, Monash University, Melbourne, Australia; 5 School of Optometry, Department of Applied Biology and Chemical Technology, Research Centre for SHARP Vision (RCSV), The Hong Kong Polytechnic University, Hong Kong, China; 6 Centre for Eye and Vision Research (CEVR), 17W Hong Kong Science Park, Hong Kong, China; 7 School of Medicine and Population Health, The University of Sheffield , Sheffield, UK; 8 Saw Swee Hock School of Public Health, National University of Singapore, National University Health System, Singapore, Singapore; 9 Laboratory of Bioinformatics and Computational Biology, Pennington Biomedical Research Center, Baton Rouge, LA, USA; 10 Department of Ophthalmology, Yong Loo Lin School of Medicine, National University of Singapore, Singapore, Singapore

## Abstract

Myopia is a global cause of vision impairment, yet its molecular mechanisms remain unclear. We applied a multi-omics approach to the retina, choroid, and sclera of guinea pigs (GPs), a model of spontaneous myopia. Integrated transcriptomic and proteomic analyses suggest a two-state molecular framework. In a myopia-prone state, the choroid and sclera exhibited widespread transcript-protein discordance, with RNA and protein changes in remodeling pathways not aligned in direction, alongside metabolic down-regulation and suppressed immune transcription, a primed yet quiescent state preceding elongation. In a spontaneous myopic state, molecular regulation becomes more concordant, with RNA and protein changing in the same direction, indicating active remodeling, whereas ribosome/translation emerged as a consistent cross-tissue signal. Integration with human myopia GWAS loci, followed by validation of CYP26A1 expression in GP ocular tissues at both transcript and protein levels, highlighted post-transcriptional regulation in the sclera and underscored translational relevance. These findings provide a molecular framework for myopia development and nominate targets in neurogenesis, synaptic remodeling, and translational control for therapeutic intervention.

## Abstract (precis)

Jiang et al apply cross-tissue dual-omics to guinea pig spontaneous myopia, revealing a primed transcript-protein discordant susceptibility state and a concordant remodeling state during myopia. Human GWAS integration and CYP26A1 validation support translational relevance and highlight remodeling and translational control.

## Introduction

Global myopia prevalence is projected to increase from ∼23% in 2000 to nearly 50% by 2050 (Holden et al, 2016). Based on this model, subsequent analyses estimate that prevalence reached ∼34% in 2020 and is expected to approach 40% by 2030 (Nemeth et al, 2021; Pan et al, 2025a). Among affected individuals, ∼2.7% develop high myopia, which significantly increases the risk of irreversible vision loss from complications such as myopic choroidal neovascularization (Wong et al, 2014). The public health burden of high and pathological myopia continues to grow, with particularly rapid increases observed in East and Southeast Asian populations (Fricke et al, 2012). A recent large-scale epidemiological study in Chinese children suggests that although the overall incidence of myopia may be stabilizing, the incidence of high myopia is still increasing (Pan et al, 2025b). This trend underscores the urgent need to understand the biological mechanisms that drive progression toward pathological states.

Although the etiology of myopia remains incompletely understood, excessive axial elongation is widely attributed to the interplay of genetic and environmental factors (Morgan et al, 2012; Tedja et al, 2019). Visual signals generated by optical defocus are thought to originate in the retina and transmit through the retinal pigment epithelium (RPE) and choroid to the sclera, ultimately leading to structural remodeling (Brown et al, 2022; Erdinest et al, 2023). Most mechanistic knowledge comes from experimentally induced models (Schaeffel et al, 1988; Hung et al, 1995; Wildsoet & Wallman, 1995; Norton et al, 2006). Although spontaneous models of myopia are available (Jiang et al, 2011; Jiang et al, 2022), they remain underexplored despite their potential to better capture naturally occurring and pathophysiologically relevant signaling cascades.

Refractive regulation depends on coordinated interactions among the retina, choroid, and sclera throughout ocular growth (Wallman & Winawer, 2004), and recent reviews have highlighted the importance of this inter-tissue communication in myopia development (Summers et al, 2021; Brown et al, 2022). However, most molecular studies have examined ocular tissues in isolation and largely in experimentally induced models (Stone et al, 2011; Wen et al, 2021; Goto et al, 2022; Ji et al, 2023; Li et al, 2025), limiting our insights into whole-eye coordination and spontaneous myopia development. Although transcriptomic and proteomic methods have been increasingly applied in myopia research (Grochowski et al, 2020; Jiang et al, 2023; Jiang et al, 2024), integrated dual-omics analyses spanning retina, choroid, and sclera remain rare, particularly in spontaneous myopia models. This leaves an important gap in understanding how molecular communication across ocular tissues may relate to susceptibility and progression under more naturally occurring conditions.

To address this limitation, an integrated transcriptomic–proteomic strategy offers the opportunity to examine molecular regulation at complementary levels across multiple ocular tissues. Because gene regulation occurs across multiple levels, including transcription, RNA processing and stability, translational efficiency, and protein turnover, changes in mRNA abundance do not necessarily translate into corresponding protein-level changes (Liu et al, 2016). When tissues are collected at a single developmental time point, this layered view may provide indirect insight into regulatory states associated with ongoing ocular growth and remodeling. In this context, transcript-protein discordance may reflect regulatory processes beyond transcription alone, including post-transcriptional buffering (Perl et al, 2017) or delayed protein accumulation (Fournier et al, 2010; Gedeon & Bokes, 2012), whereas concordant regulation across both layers is more consistent with coordinated execution of biological programs (Lackner et al, 2012). Although time-resolved studies are required to distinguish these possibilities definitively, this framework provides a useful basis for interpreting molecular states associated with susceptibility versus those linked to more active tissue remodeling.

Here, we implemented an integrated multi-omics approach to profile transcriptomic and proteomic signatures across the retina, choroid, and sclera in albino hyperopic (AH), spontaneously albino myopic (AM), and pigmented hyperopic (PH) guinea pigs (GP). Comparison of AH and PH animals, which share a hyperopic state but differ in pigmentation background and susceptibility, was used to identify molecular features associated with myopia predisposition. Comparison of AM and AH littermates was used to identify pathways associated with spontaneous myopia progression. By integrating transcriptomic and proteomic datasets, applying pathway enrichment analyses, and examining patterns of concordance and discordance across ocular tissues from the same eye, this dual-comparison design captures both susceptibility-related and myopia-associated states within the same species. Building on evidence for population-specific differences in myopia prevalence and progression (Hagen et al, 2018; Luong et al, 2020), this framework improves biological interpretability and highlights molecular cascades with potential translational relevance.

## Results

We first established cross-tissue transcriptomic and proteomic datasets from the retina, choroid, and sclera at postnatal day 14 (P14) for two complementary comparisons: albino versus pigmented hyperopic animals (AH versus PH), to examine molecular features associated with susceptibility, and spontaneously myopic albinos versus their hyperopic littermates (AM versus AH), to capture molecular changes associated with spontaneous myopia. For each comparison, we characterized differentially expressed genes (DEGs) and proteins (DEPs), examined their overlap across tissues, and assessed transcript-protein concordance within each tissue, followed by pathway enrichment analyses to define shared and tissue-specific biological programs. This integrative framework enabled susceptibility-associated signatures to be distinguished from progression-associated changes: susceptibility was marked by broad transcriptional alterations not yet consistently reflected at the protein level, suggesting a primed regulatory state, whereas progression showed greater transcript-protein alignment, consistent with more active execution of remodeling programs.

### Confirmed refractive phenotypes of albino and pigmented GPs at P14

**Figure 1.**
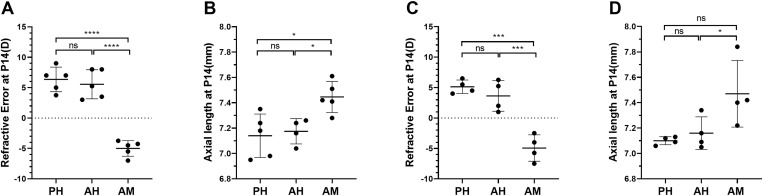
Refractive and axial phenotypes of the pre-selected omics cohort at postnatal day 14. **(A, B, C, D)** Cycloplegic refractive error and (B, D) axial length in AH, AM, and PH guinea pig (GP) selected for transcriptomic ((A, B); n = 4) and proteomic ((C, D); n = 5) analysis. Each symbol represents one animal; bars show mean ± SD. Ordinary one-way ANOVA with Tukey’s multiple-comparisons test was used. Ns, Not Significant, *P < 0.05; ***P < 0.001; ****P < 0.0001. These comparisons are presented to confirm the phenotypic separation of the selected subset used for omics profiling.

We first characterized the ocular phenotypes of the GPs selected for transcriptomic (Fig 1A and B) and proteomic (Fig 1C and D) analyses. As a model for molecular susceptibility, we compared hyperopic eyes from pigmented (PH) and albino (AH) animals, which showed comparable refraction and axial length. As a model for spontaneous myopia, we compared myopic albino eyes (AM) with hyperopic albino controls (AH) and, as expected, found significant differences in both refractive error and axial length. These phenotypic contrasts provided the foundation for interpreting subsequent multi-omics signatures in relation to myopia susceptibility versus spontaneous myopia.

### Global transcriptomic and proteomic landscapes across ocular tissues

**Figure 2.**
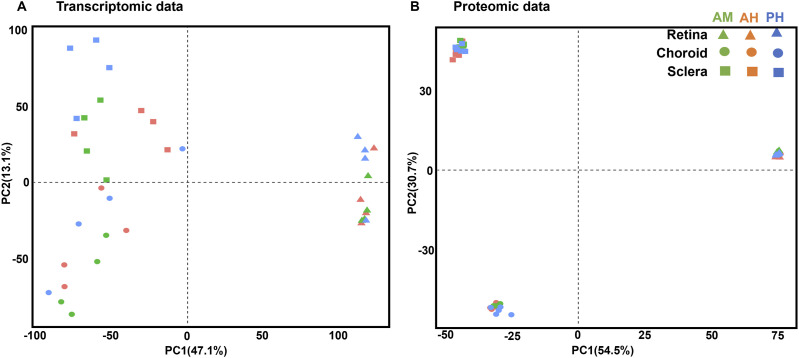
PCA of tissue clustering in transcriptomic (A) and proteomic (B) datasets. PC1–PC2 plots show clear tissue-specific clustering based on mRNA ((A); n = 4) and protein ((B); n = 5) profiles from pigmented (PH, blue) and albino GPs (AM, green; AH, red). Retina, choroid, and sclera are represented by triangles, circles, and squares, respectively.

After filtering out low-expression genes (average counts ≤5) and accounting for batch effects, 16,831, 17,320, and 17,515 genes were detected in the retina, choroid, and sclera, respectively; identical filtering and differential expression criteria, including Benjamini–Hochberg false discovery rate (FDR) correction and log2 fold-change thresholds, were applied across all tissues to ensure comparability and highlight robust signals for downstream analysis. Similarly, 8,322, 8,714, and 8,074 proteins were identified in these tissues. Principal components analysis (PCA) of normalized transcriptomic and proteomic datasets showed robust segregation of samples by tissue type (Fig 2A and B). In the transcriptomic data, PC1 (47.1%) distinguished retina from choroid and sclera, whereas PC2 (13.1%) further separated choroid from sclera. Proteomic data showed a similar pattern, with PC1 (54.5%) separating retina from the other tissues and PC2 (30.7%) differentiating choroid from sclera. Although tissue identity was the dominant source of variance, the transcriptomic PCA further indicated greater within-tissue dispersion in choroid and sclera than in retina. All samples were segregated primarily by tissue type, with retina clearly separated from choroid and sclera along PC1 (Fig 2A), the dominant axis of variation. Greater within-tissue dispersion was observed among choroid and sclera samples, particularly along PC2 (Fig 2A), likely reflecting biological heterogeneity together with minor technical and/or cross-layer variation. Importantly, this dispersion did not compromise overall tissue-level separation or downstream analyses. We further note that the greater dispersion in choroid and sclera may have contributed, at least in part, to the larger number of DEGs identified in these tissues under the uniform thresholds applied across comparisons, and this possibility should be considered when interpreting differences in DEG counts across tissues.

### Distinct molecular profiles in AH and PH despite a shared hyperopic state

We next examined molecular differences between AH and PH across the retina, choroid, and sclera at P14, despite their shared hyperopic refractive state.

### Gene- and protein-level differences in the retina, choroid, and sclera

**Figure 3.**
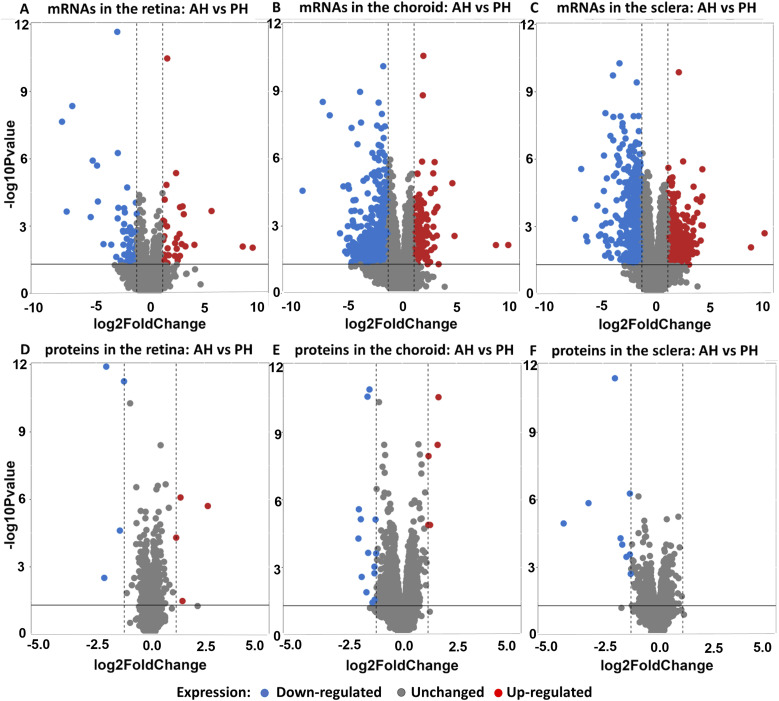
Volcano plots of DEGs (A, B, C) and DEPs (D, E, F) in AH tissues compared with PH controls. Volcano plots show gene and protein expression changes in the retina (A, D), choroid (B, E) and sclera (C, F). Blue and red dots indicate significantly down- and up-regulated DEGs/DEPs, respectively, whereas gray dots are not significant. Significance thresholds are ± 0.263 Log_2_ fold change (1.2-fold) for the vertical lines and –Log_10_ *P*-value of 1.301 (*P* ≤ 0.05) for the horizontal line.

Volcano plots summarized tissue-specific transcriptomic (Fig 3A–C) and proteomic (Fig 3D–F) differences between AH and PH samples. For a descriptive comparison of transcriptomic and proteomic signal extent across tissues, we used a permissive threshold of absolute fold-change ≥1.2 and nominal *P* ≤ 0.05. Under this criterion, transcriptomic changes were more extensive than proteomic changes. This threshold was used for visualization and cross-platform comparison rather than for primary statistical inference. The retina showed relatively few differences (Fig 3A, 100 DEGs, 67 down-regulated; Fig 3D, 8 DEPs, 4 down-regulated), whereas the choroid and sclera displayed nearly tenfold more DEGs (Fig 3B, choroid: 920, 717 down-regulated; Fig 3C, sclera: 1,051, 472 down-regulated). In contrast, proteomic alterations in these tissues were limited (Fig 3E, choroid: 19 DEPs, 14 down-regulated; Fig 3F, sclera: 9 DEPs, all down-regulated) (Table S1). This pronounced difference between transcriptomic and proteomic signals may reflect a combination of biological regulation and methodological differences in sensitivity, coverage, and dynamic range between RNA sequencing and mass spectrometry-based proteomics. Within this context, the extensive transcriptional changes observed in the choroid and sclera are interpreted as compatible with a primed regulatory state, with relatively limited translation into stable protein-level differences at this stage.

Table S1. Significant genes and proteins identified by volcano plot analysis in AH versus PH across retina, choroid, and sclera.

We next compared overlaps among DEGs and DEPs across the three tissues using Venn analysis for up- and down-regulated genes (Tables 1 and S2), as a descriptive strategy to identify shared or recurrent molecular features across ocular tissues rather than to infer direct inter-tissue signal propagation. To ensure fair comparison, we restricted the analysis to the top 1% of DEGs and DEPs in each category, ranked by the product of nominal *P*-value and log_2_ fold-change.

**Table 1.** Number of overlapping hits^a^ within different tissues among the top 1% hits.

​	​	AH versus PH			
​	​	C versus R	C versus S	R versus S	Common in R-C-S
DEG	Up-regulated	24	30	19	12
	Down-regulated	10	31	13	7
DEP	Up-regulated	2	6	7	1
	Down-regulated	6	20	7	3

a A complete list of overlapping genes and proteins, including unannotated loci, is provided in Table S2.

Table S2. Venn analysis of tissue-specific and shared dysregulated genes and proteins in AH versus PH.

Among up-regulated DEGs, 12 genes were shared across all three tissues, including *UbiA prenyltransferase domain-containing* 1 (*UBIAD1*), *zinc finger protein 667* (*ZNF667*), *Aurora kinase C* (*AURKC*), and *thioredoxin domain-containing 11* (*TXNDC11*), along with several unannotated loci. In total, 24 genes were up-regulated in retina–choroid, 30 in choroid–sclera, and 19 in retina–sclera comparisons. Seven down-regulated DEGs were shared across all tissues, including *Cytochrome P450 family 1 subfamily A member 1* (*CYP1A1*), *ATPase H*^*+*^ *transporting V0 subunit D2* (*ATP6V0D2*), *Kallikrein-related peptidase 4* (*KLK4*), and *Kynurenine 3-monooxygenase* (*KMO*), and were part of the 10, 31, and 13 genes commonly down-regulated in the respective tissue pairs.

For DEPs, one up-regulated protein, premelanosome protein (PMEL), was consistently shared across all three tissues, whereas 2, 6, and 7 proteins were commonly up-regulated in the three pairwise comparisons. Three down-regulated proteins, spermidine/spermine N^1^-acetyltransferase family member 2 (SAT2)*,* glutathione S-transferase kappa 1 (GSTK1), and one other, were shared by all tissues, with 6, 20, and 7 down-regulated proteins shared by the tissue pairs. A complete list of overlapping genes and proteins, including unannotated loci, is provided in Table S2. Notably, some overlapping features in the AH versus PH comparison, such as PMEL, are likely to reflect pigmentation-related background differences rather than susceptibility-specific mechanisms.

Although the proportion of shared features across tissues was broadly similar between transcriptomic and proteomic datasets, the absolute number of overlapping transcripts was greater, facilitating clearer identification of shared regulatory programs at the transcriptional level. By contrast, proteomic overlap was more limited in absolute terms, likely reflecting both lower feature counts and reduced detection sensitivity.

Finally, we assessed concordance between DEGs and DEPs within each tissue. No concordant up-regulated hits were detected in any tissue, whereas concordant down-regulated hits were limited to the choroid and sclera. In the choroid, four gene-protein pairs showed concordant down-regulation—*SLC6A11/SLC6A11 *(solute carrier family 6 member 11), *SLC6A1/SLC6A1 *(solute carrier family 6 member 1), *NEFL/NEFL* (neurofilament light chain), and *GSTK1*/GSTK1, whereas in the sclera, three concordant down-regulated pairs were identified—*troponin T3*/troponin T3, *TNNT3*/TNNT3 (fast skeletal type), *MYH1*/MYH1 (myosin heavy chain 1), and *GSTK1*/GSTK1 (Table 2). These findings indicate limited tissue-specific transcript-protein concordance, with overlapping down-regulated molecules in the choroid enriched for neuronal-associated components and those in the sclera, including contractile-related proteins, whereas GSTK1 was shared by both tissues. No transcript-protein overlap was detected in the retina. Overall, gene-protein concordance at P14 was limited across tissues, particularly in the retina, indicating that transcriptomic changes were not yet broadly mirrored at the protein level and motivating pathway-level analysis to assess whether higher order biological programs showed greater consistency than individual features.

**Table 2.** Number of overlapping hits between transcriptomic and proteomic.

​	AH versus PH		
​	Retina	Choroid	Sclera
Up-regulated	0	0	0
Down-regulated	0	4	3

### Pathway-level signatures across ocular tissues

Table 3 summarizes the number of significantly up-regulated and down-regulated pathways identified in each omics dataset at adjusted *P* ≤ 0.05, with full details in Table S3. Transcriptome enrichment indicated a predominance of down-regulated pathways in AH relative to PH, whereas proteomics enrichment was more balanced between up-regulation and down-regulation.

**Table 3.** The number of significantly up-regulated or down-regulated pathways in AH and PH.

AH versus PH	Transcriptomics-based GSEA		Proteomics-based GSEA	
	Up-regulated	Down-regulated	Up-regulated	Down-regulated
Retina	1	25	11	8
Choroid	5	14	13	22
Sclera	9	36	22	21

Table S3. Significant KEGG signaling pathways at gene and protein levels in AH versus PH across retina, choroid, and sclera.

**Figure 4.**
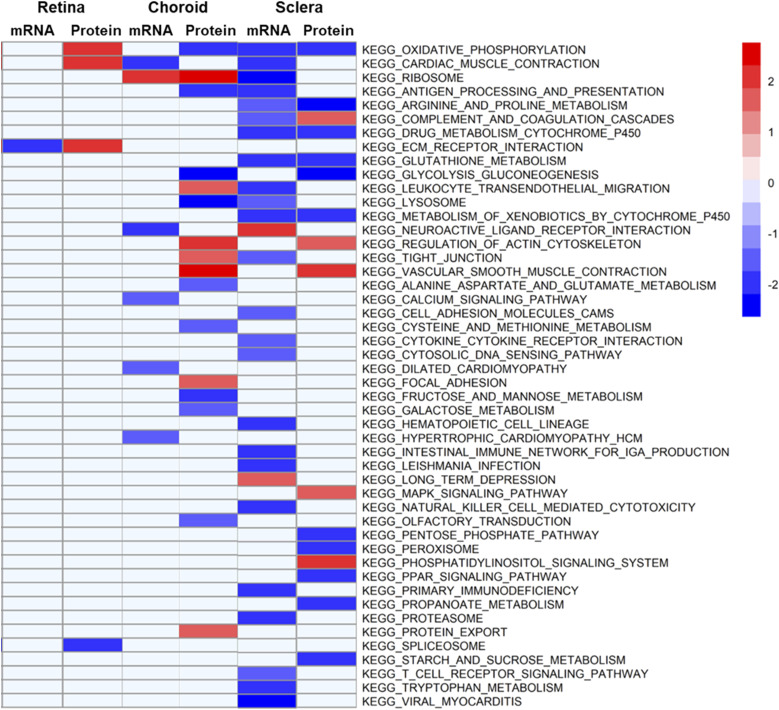
Comparison of top significantly enriched pathways between AH and PH across transcriptomic and proteomic datasets. Pathway activity is shown for retina, choroid, and sclera. Red color represents up-regulated pathways, and blue color represents down-regulated pathways.

A comparison of the top significantly enriched pathways (adj *P* ≤ 0.05) from both transcriptomic and proteomic datasets across omics layers and tissues is visualized in the heatmap (Fig 4), with normalized enrichment scores and corresponding FDR or adjusted *P*-values provided in Table S4. We highlight three major categories of pathway signatures.

Table S4. Statistical metrics for cross-tissue and cross-omics pathway enrichment in AH versus PH.

First, several pathways exhibited discordant regulation between transcriptomic and proteomic layers. For example, the ECM receptor interaction pathway in the retina was down-regulated at the transcript level but up-regulated at the protein level. Similarly, complement and coagulation cascades in the sclera were down-regulated at the mRNA level yet up-regulated at the protein level. These mismatches are consistent with post-transcriptional regulation that may buffer or amplify remodeling responses at the protein level.

Second, a larger set of pathways showed strong concordance, with pathway-level agreement exceeding gene-level overlap. This likely reflects aggregation of coordinated but individually modest changes across functionally related genes and proteins, making pathway-level convergence a more robust readout of shared regulation. Ribosome signaling emerged as a consistent signal, being up-regulated across the choroid, whereas the sclera exhibited a cluster of concordantly down-regulated metabolic pathways, including oxidative phosphorylation, arginine–proline metabolism, glutathione metabolism, and cytochrome P450-related processes. This pattern indicates a coordinated metabolic reprogramming in the sclera, with suppression of mitochondrial energy production and redox defense aligning with the down-regulation of GSTK1 observed at the individual protein level.

Finally, in the sclera, several immune-related pathways were consistently down-regulated at the transcriptomic level but showed no significant change at the proteomic level, including those related to T-cell receptor signaling, natural killer cell–mediated cytotoxicity, cytokine–cytokine receptor interaction, and antigen processing and presentation. This selective suppression at the transcriptional level, without proteomic reinforcement, suggests a state of immune quiescence rather than outright immune loss, potentially maintaining tissue homeostasis under altered metabolic conditions.

Taken together, these pathway-level signatures suggest that AH animals harbor a distinct molecular milieu characterized by (i) transcript-protein discordance in remodeling-related pathways, (ii) coordinated down-regulation of scleral metabolic activity, and (iii) transcriptional suppression of immune signaling without proteomic reinforcement.

### Early molecular signatures distinguishing AM and AH at P14

After examining molecular signatures associated with susceptibility in the AH versus PH comparison, we next compared AM and their counterparts AH to identify molecular features associated with the myopic state at P14. Overall, relatively few DEGs and DEPs were detected in each tissue, with the sclera showing the most extensive transcriptomic alterations. Subsequent pathway enrichment and cross-tissue analyses highlighted both concordant and discordant molecular programs, offering insight into molecular processes linked to spontaneous myopia remodeling.

### Gene- and protein-level alterations in retina, choroid, and sclera

**Figure 5.**
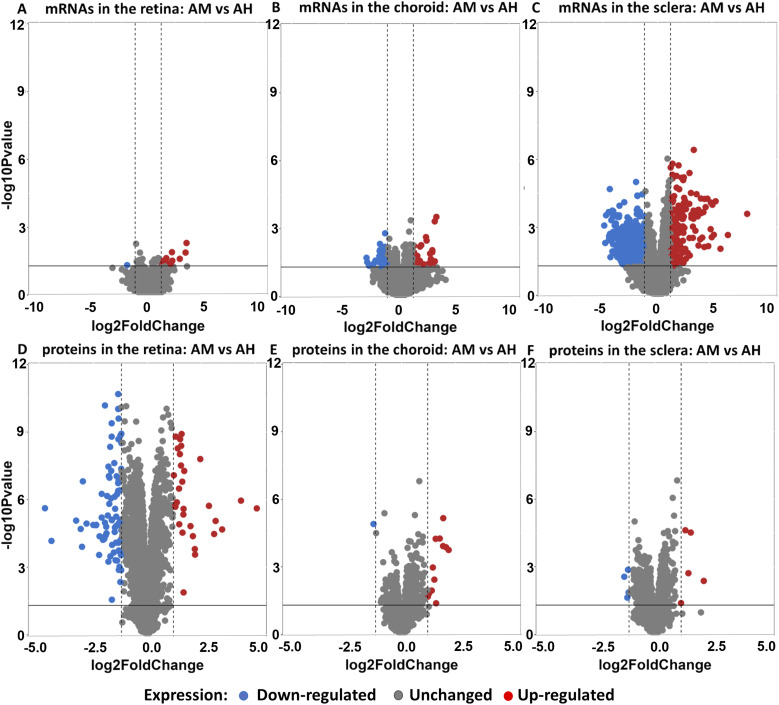
Volcano plots of DEGs (A, B, C) and DEPs (E, F) in AM compared with AH tissues. **(A, B, C, D, E, F)** Panels show expression changes in the retina (A, D), choroid (B, E) and sclera (C, F). Blue and red dots represent significantly down-regulated and up-regulated DEGs/DEPs, respectively, whereas gray dots indicate non-significant changes. Vertical dashed lines denote the fold-change cutoff (±0.263 Log_2_, equivalent to 1.2-fold), and the horizontal dashed line indicates the significance threshold (−Log_10_ *P*-value = 1.301, corresponding to *P* < 0.05).

Volcano plots summarized transcriptomic and proteomic differences between AM and AH samples across the retina, choroid, and sclera (Fig 5A–F), with full DEG and DEP lists provided in Table S5. Using the threshold of absolute fold-change ≥1.2 and nominal *P* ≤ 0.05, the retina exhibited relatively few transcriptomic changes (14 DEGs, 1 down-regulated) but substantial proteomic alterations (111 DEPs, 80 down-regulated). The choroid showed a modest number of DEGs (62, 30 down-regulated) alongside 13 DEPs (1 down-regulated), whereas the sclera displayed extensive transcriptomic remodeling (729 DEGs, 551 down-regulated) with comparatively few proteomic shifts (9 DEPs, 4 down-regulated). These results highlight a pronounced tissue-specific imbalance between transcriptomic and proteomic alterations, with the sclera showing the strongest transcriptional signal.

Table S5. Significant genes and proteins identified by volcano plot analysis in AM versus AH across retina, choroid, and sclera.

We next applied the same descriptive Venn-based strategy used for the AH versus PH comparison to the AM versus AH comparison. Analysis of the top 1% of DEGs (ranked by nominal *P* × log FC; Tables 4 and S6) revealed limited convergence across tissues. Among up-regulated genes, only two—Zic family member 3 (*ZIC3*), a transcription factor implicated in early developmental patterning, and betacellulin (*BTC*), an EGF-like growth factor—were shared across all three tissues, suggesting broad activation of developmental and growth-related programs. Additional overlaps were largely pairwise, with 10, 33, and 3 genes shared between retina–choroid, choroid–sclera, and retina–sclera comparisons, respectively. No down-regulated genes were common to all tissues; however, subsets such as Pre-B lymphocyte 3 (*VPREB3*), linked to B-cell receptor signaling, and ENSCPOG00000034629 (uncharacterized) appeared in multiple tissue pairs, hinting at selective suppression of immune-related functions.

**Table 4.** Number of overlapping hits within different tissues among the top 1% hits.

​	​	AM versus AH			
​	​	C versus R	C versus S	R versus S	Common in R-C-S
DEG	Up-regulated	10	33	3	2
	Down-regulated	14	8	2	0
DEP	Up-regulated	1	14	1	0
	Down-regulated	2	19	2	0

Table S6. Venn analysis of tissue-specific and shared dysregulated genes and proteins in AM versus AH.

At the protein level, overlap was similarly sparse. No DEPs were shared across all tissues, but pairwise comparisons identified proteins such as nuclear transcription factor Y subunit alpha (NFYA, C versus R) and signal peptidase complex subunit (SPCS1, R versus S), which were consistently up-regulated in specific tissue pairs, and zinc finger and BTB domain-containing 39 (ZBTB39), RNA polymerase I transcription factor RRN3 (RRN3), and other ribosome/translation-related proteins, which were down-regulated across more than one tissue. These findings suggest that, although progression to spontaneous myopia involves tissue-specific molecular remodeling, a few developmental regulators (ZIC3, BTC) and transcriptional/translation factors (NFYA, RRN3) may represent recurrent regulatory nodes across ocular tissues. Finally, direct comparisons of DEGs and DEPs within each tissue revealed no concordant regulation between transcriptomic and proteomic datasets in the AM versus AH contrast, highlighting the importance of pathway-level analyses for identifying broader biological patterns.

### Pathway-level signatures across ocular tissues

Table 5 summarizes the numbers of significantly up- and down-regulated pathways identified in each omics dataset (adj *P* ≤ 0.05; full results in Table S7). In contrast to the AH versus PH comparison, AM versus AH samples displayed a more balanced distribution of up-regulated and down-regulated pathways at the transcriptomic level. At the proteomic level, the retina and choroid also showed relatively balanced regulation, whereas the sclera was dominated by down-regulated pathways.

**Table 5.** The number of significantly up- and down-regulated pathways in AM and AH.

AM versus AH	Transcriptomics-based GSEA		Proteomics-based GSEA	
	Up-regulated	Down-regulated	Up-regulated	Down-regulated
Retina	4	13	2	0
Choroid	8	2	13	16
Sclera	9	6	9	40

Table S7. Significant KEGG signaling pathways at gene and protein levels in AM versus AH across retina, choroid, and sclera.

**Figure 6.**
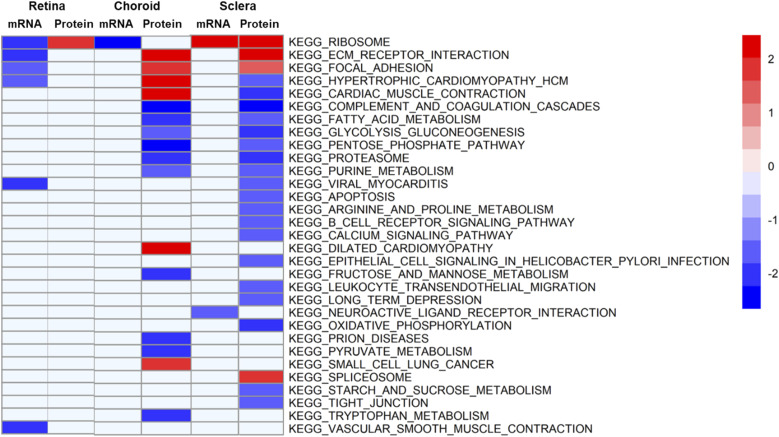
Comparison of top significantly enriched pathways between AM and AH across transcriptomic and proteomic datasets. Pathway activity is shown for retina, choroid and sclera. Red colours represent up-regulated pathways, and blue colours represent down-regulated pathways.

Heatmaps in Fig 6 provide a comparative overview of the top enriched pathways (adj *P* ≤ 0.05) across transcriptomic and proteomic datasets within the three tissues. Concordance between transcriptomic and proteomic enrichment was generally limited, with the ribosome pathway emerging as the most consistent cross-tissue signal. This pathway reached statistical significance in all datasets except the choroid proteome, and its regulation was tissue-specific: up-regulated in the sclera, down-regulated in the choroid, and discordant in the retina (down-regulated at the transcript level but up-regulated at the protein level). These patterns point toward enhanced protein synthesis in the sclera, suppressed translational capacity in the choroid, and potential post-transcriptional or compensatory regulation in the retina.

Structural pathways, including ECM–receptor interaction and focal adhesion, were consistently up-regulated at the protein level in the choroid and sclera but down-regulated in the retina at the transcript level. Functionally related cardiac muscle–associated pathways (e.g., hypertrophic cardiomyopathy and cardiac muscle contraction) showed tissue-dependent proteomic regulation, being up-regulated in the choroid but down-regulated in the sclera.

In contrast, several metabolic and proteostasis-related pathways were broadly suppressed at the protein level in both the choroid and sclera. These included fatty acid metabolism, glycolysis/gluconeogenesis, the pentose phosphate pathway, the proteasome, and purine metabolism, suggesting reduced metabolic activity and proteostasis capacity in these tissues during spontaneous myopia progression.

In summary, AM versus AH comparisons revealed tissue-specific regulation of structural, metabolic, and proteostasis pathways, with the ribosome pathway emerging as the most consistent yet context-dependent signal. These heterogeneous molecular responses raise the question of whether the implicated genes bear translational relevance to human myopia. Normalized enrichment scores and corresponding FDR or adjusted *P*-values of these pathways were provided in Table S8.

Table S8. Statistical metrics for cross-tissue and cross-omics pathway enrichment in AM versus AH.

### Translational relevance: GP genes linked to human myopia

**Figure 7.**
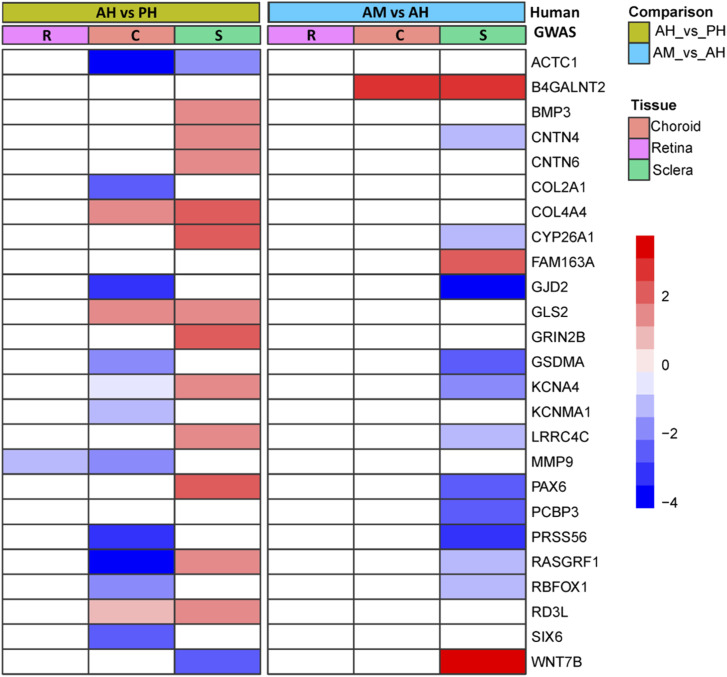
Cross-species overlap between human GWAS loci and GP DEGs across ocular tissues. Heatmap showing overlap between GP DEGs and human myopia-associated loci. The left panel depicts DEGs from AH versus PH comparison (susceptibility) and the right panel shows DEGs from AM versus AH (spontaneous myopia). Rows represent genes, and color intensity represents log fold change (red, up-regulated; blue, down-regulated). Columns are organized by tissue: retina (pink), choroid (salmon), and sclera (green).

We, therefore, assessed whether genes linked to myopia susceptibility and spontaneous myopia in GPs overlap with loci identified in human myopia GWAS, thereby highlighting candidates of potential translational importance. Key findings are summarized in Fig 7, which distinguishes susceptibility-related genes from those associated with spontaneous myopia progression, providing a cross-species bridge to human myopia genetics. Human myopia GWAS hits were more broadly represented among GP susceptibility genes in both the choroid and sclera, whereas overlaps with spontaneous myopia genes were concentrated in the sclera. Notably, several scleral genes, including differential expression of contactin 4 (*CNTN4*), *CYP26A1*, Potassium voltage-gated channel subfamily A member 4 (*KCNA4*), leucine-rich repeat containing 4C (*LRRC4C*), paired box 6 (*PAX6*), and RAS protein-specific guanine nucleotide-releasing factor 1 (*RASGRF1*), switched from up-regulation in susceptibility to down-regulation in spontaneous myopia, underscoring their context-dependent roles.

### Validation of *CYP26A1* expression

**Figure 8.**
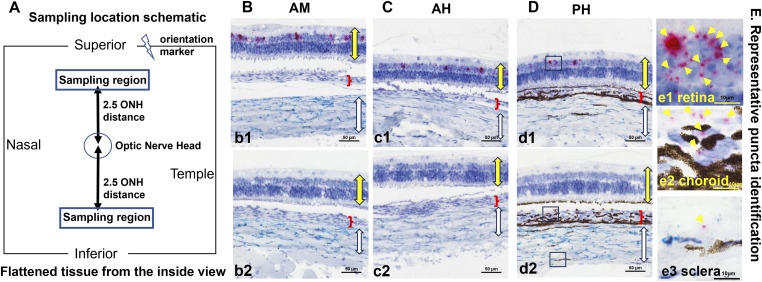
Regional distribution and exploratory assessment of condition-dependent *CYP26A1* expressions. **(A)** Sampling scheme. In the left eye, sections for RNAscope were collected at ∼2.5 optic nerve head (ONH) diameters from the ONH center in the superior and inferior regions. **(B, C, D)** Representative RNAscope images showing *CYP26A1* mRNA (red puncta) in the retina (yellow double arrows), choroid (red brackets), and sclera (white double arrows) under spontaneous myopia ((B), AM), myopia-prone ((C), AH), and myopia-resistant ((D), PH) states. For each group, the upper panel shows the superior region (b1, c1, and d1) and the lower panel shows the inferior region (b2, c2, and d2). **(E)** Representative examples of *CYP26A1* RNAscope puncta identification in the retina (e1), choroid (e2), and sclera (e3). Boxed regions are shown at higher magnification (100×), and yellow arrows indicate counted puncta used for exploratory density measurement. n = 1 animal per group (5 wk old). *CYP26A1* signal was observed mainly in the retina, with lower signal in the choroid, and sparse signal in the sclera. Images are representative of the analyzed regions and are shown for illustrative purposes only. A corresponding exploratory semi-quantitative assessment is provided in Table 6. Scale bars, 50 μm.

We performed exploratory validation of *CYP26A1* expression at both the transcript and protein levels. Representative RNAscope images from the superior and inferior regions equally distant to the optic nerve head (Fig 8A) suggested region-dependent *CYP26A1* signal, with a stronger signal in the retina (Fig 8B–b1, C–c1, and D–d1), a lower signal in the RPE/choroid (Fig 8B–D), and a sparse signal in the sclera (Fig 8B–D); corresponding exploratory puncta-density measurements from the analyzed sections are summarized in Table 6. In the susceptibility condition (Fig 8C, AH), choroidal and scleral *CYP26A1* mRNA appeared higher than in controls (Fig 8D, PH), whereas retinal levels showed little difference. In the spontaneous myopia state (Fig 8B, AM), retinal and choroidal *CYP26A1* mRNA appeared elevated relative to AH, whereas scleral levels remained unchanged. These findings broadly aligned with the transcriptomic results in the sclera.

**Table 6.** Exploratory RNAscope puncta-density measurements (puncta per 100,000 μm^2^).

Location	Tissue	AM	AH	PH
Superior	Retina	382	261	302
	RPE/choroid	71	53	45
	Sclera	8	4	0
Inferior	Retina	116	116	148
	RPE/choroid	75	66	17
	Sclera	4	9	1
Sum	Retina	250	204	226
	RPE/choroid	73	60	31
	Sclera	12	13	1

See Fig 8E for representative examples of puncta identification.

**Figure 9.**
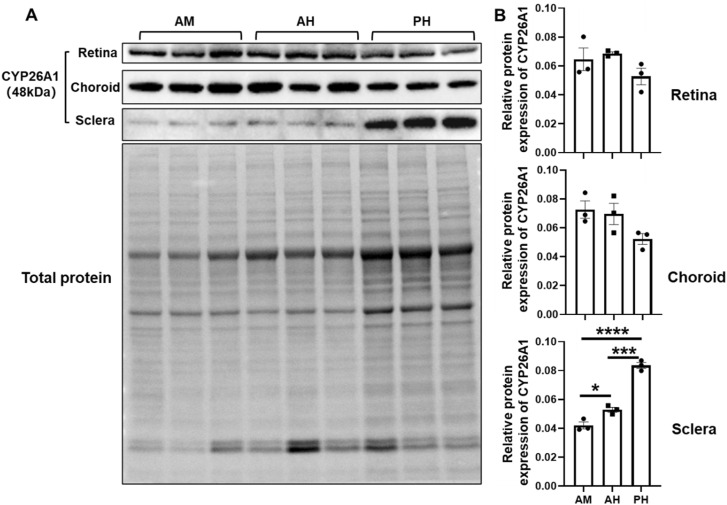
Tissue-specific assessment of CYP26A1 expression. **(A)** Representative Western blots showing CYP26A1 expression in ocular tissues, with corresponding total protein staining visualized using the No-Stain reagent for post-transfer normalization. Full blot images and the corresponding total protein staining are provided in Fig S1A–C. **(B)** Densitometric semi-quantification of CYP26A1, presented as relative fold change after normalization to the total protein signal of the corresponding lane on the same membrane (mean ± SD). No significant changes were observed in the retina or choroid, whereas scleral CYP26A1 was decreased in both the myopia-prone and spontaneous myopia groups. Data were obtained from a separate cohort of animals (n = 3 per group, 5 wk old). Statistical significance was assessed by one-way ANOVA followed by Tukey’s post hoc test; *P < 0.05, ***P < 0.001.

**Figure S1.**
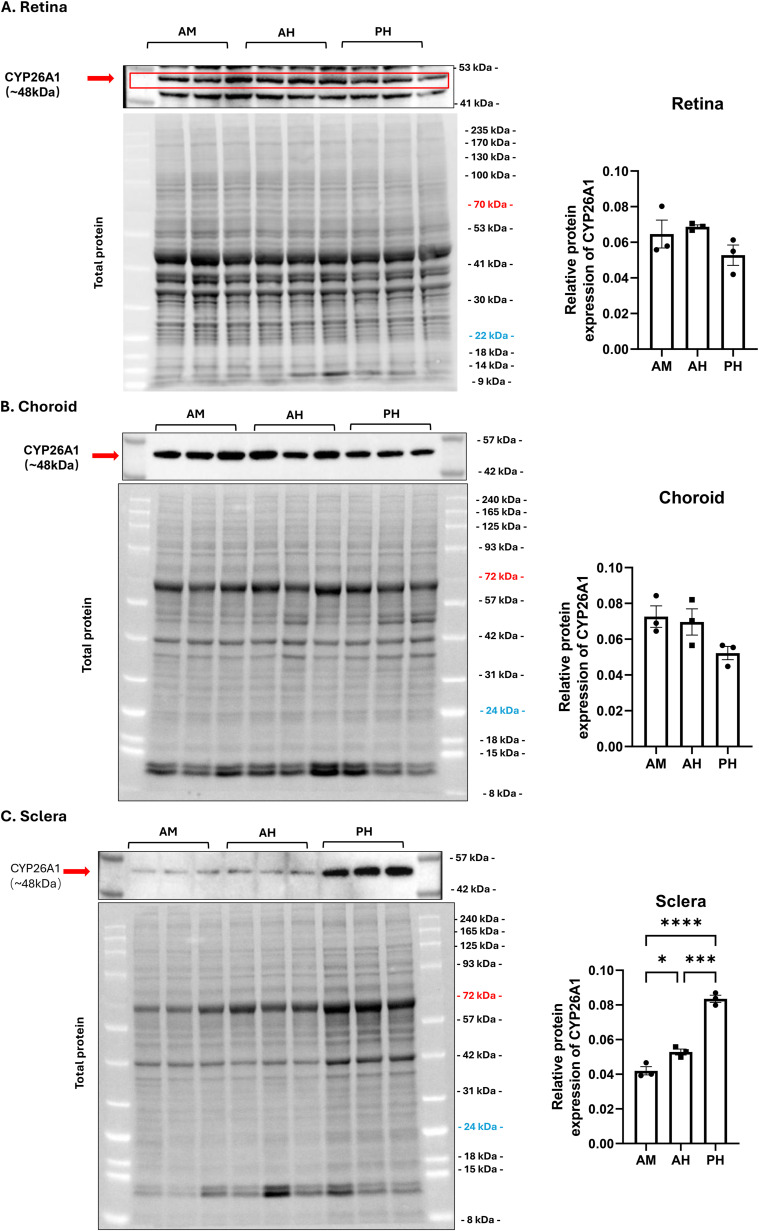
Representative total protein normalization images and corresponding CYP26A1 immunoblots across ocular tissues.

Western blot analysis in a separate cohort (Fig 9A) provided partial support for these tissue-specific patterns. As shown by semi-quantitative densitometric (Fig 9B), retinal CYP26A1 protein expression, presented as relative fold change after normalization to total protein, did not differ significantly among groups, consistent with the transcript-level data. In the choroid, CYP26A1 protein expression showed a non-significant trend toward higher relative expression in albino eyes. In contrast, scleral CYP26A1 protein expression was significantly decreased in both AH and AM relative to PH, opposite to the corresponding mRNA pattern. Full blot images and the corresponding total protein staining are provided in Fig S1A–C. Together, these exploratory data suggest broadly concordant transcript-protein patterns for CYP26A1 in the retina and RPE/choroid but an apparent discordance in the sclera.

## Discussion

This study is, to our knowledge, the first to integrate high-throughput transcriptomic and proteomic profiling across the retina, choroid, and sclera to investigate molecular programs associated with myopia susceptibility and spontaneous myopia in an animal model with naturally occurring refractive error. Our dual-omics design enabled cross-tissue comparison of molecular changes across ocular layers and identified both recurrent pathways, including translation/ribosome signaling and ECM remodeling, and tissue-specific programs relevant to myopia. An important advantage of this approach is that it helps distinguish molecular signatures associated with susceptibility from those linked to progression. In myopia-prone eyes, widespread transcriptomic changes in choroid and sclera were not yet consistently reflected at the protein level, consistent with a primed regulatory state and possible post-transcriptional buffering. In contrast, spontaneous myopia showed greater transcript-protein concordance, consistent with more active remodeling.

To place these findings in a broader context, we organized the discussion around three themes: molecular features linked to myopia susceptibility, protein-level regulation in spontaneous myopia, and concordance with human myopia GWAS loci. Myopia susceptibility, as modeled here, was associated with coordinated changes in ECM-related, immune-related, and metabolism-related pathways across ocular tissues. Although the present data do not establish a mechanism, these patterns may mark a molecular environment relevant to later ocular growth regulation.

In the retina, ECM-receptor interaction pathways showed marked transcript-protein discordance: broad transcriptional down-regulation of collagens, laminins, and integrins contrasted with proteomic up-regulation of selected laminin and proteoglycan components. Although the retina is predominantly neural, ECM and basement-membrane proteoglycan systems are well represented at retinal structural interfaces, supporting the relevance of ECM-associated pathway shifts in this tissue (Keenan et al, 2012). This pattern suggests that, despite reduced ECM-related transcripts, key structural proteins may be maintained through selective translation, differential turnover, or protein stabilization (Aisenbrey et al, 2006). Such regulation may help preserve basement-membrane integrity and mechanotransductive responsiveness in the retina.

In parallel with the retinal findings, the choroid showed susceptibility-associated changes in neurochemical, redox, and contractile-associated pathways. Down-regulation of the GABA transporters SLC6A11 and SLC6A1 indicates altered GABA-related signaling, consistent with previously reported strain-dependent differences in scotopic ERG responses (Jiang et al, 2025). Reduced NEFL, TNNT3, and MYH1 may reflect diminished contractile or cytoskeletal responsiveness. Shared down-regulation of GSTK1, in addition, indicates increased oxidative vulnerability. Together, these changes support the view that the choroid may serve as an intermediate regulatory tissue in which neural-associated, metabolic, redox, and biomechanical influences converge before overt elongation (Tedja et al, 2018).

In the sclera, broad transcriptomic down-regulation of immune-related pathways contrasted with proteomic up-regulation of complement and coagulation cascades, again indicating marked transcript-protein discordance. Given prior evidence implicating cytokines and immune pathways in myopia development in both humans (Yuan et al, 2019; Jiang et al, 2024) and animal models (Lin et al, 2016; Xiao et al, 2025), one interpretation is that transcriptional immune quiescence may coexist with selective protein-level activation of complement-related pathways. Such a state could permit remodeling-related immune surveillance, although limiting excessive inflammatory or cross-linking responses (Riddell & Crewther, 2017; Zeng et al, 2023), thereby favoring a more compliant scleral matrix, a biomechanical condition linked to axial elongation.

Consistent with these tissue-specific differences, the choroid and sclera showed complementary metabolic features, with ribosome up-regulation in the choroid suggesting increased translational capacity, whereas down-regulation of oxidative phosphorylation, arginine–proline metabolism, and cytochrome P450 pathways in the sclera indicated a relatively hypometabolic state.

Together, these findings support a cross-tissue framework in which retinal structural signaling, choroidal neurochemical and metabolic integration, and scleral immune-metabolic remodeling are each associated with myopia susceptibility. Although some differences are also likely to reflect pigmentation biology and broader strain-specific background effects, circadian influences, including potential effects of melatonin signaling, cannot be fully excluded in the PH versus AH comparison, even though this risk was minimized by standardized housing conditions and a restricted tissue-collection window. The overall pattern suggests that susceptibility involves coordinated but tissue-specific molecular states across adjacent ocular layers. Subsequent divergence in eye growth, however, is likely to depend not only on these baseline molecular features but also on physiological responses that emerge during the induction phase.

The transition from widespread transcript-protein discordance in the susceptibility state to greater concordance in spontaneous myopia is consistent with a shift from regulatory priming toward more active molecular execution. In this framework, concordance across omics layers is more consistent with engagement of downstream remodeling processes, whereas discordance is more compatible with buffering or preparatory regulatory states. In spontaneously myopic eyes, most pathways were regulated in the same direction across the retina, choroid, and sclera, suggesting a more coordinated phase of tissue remodeling associated with myopia.

The ribosome pathway displayed the clearest multi-tissue divergence, revealing distinct translational dynamics across ocular layers. Ribosomal genes were down-regulated in the retina and choroid but up-regulated in the sclera, indicating sclera-specific transcriptional activation of protein synthesis. At the protein level, ribosomal components were up-regulated in both the retina and sclera, suggesting enhanced translational efficiency or protein stabilization in the retina despite transcriptional suppression. Shared down-regulation of ribosome-associated regulators, such as *RRN3* and *ZBTB39*, further points to common translational control nodes. Elevated ribosomal activity in the sclera likely supports ECM remodeling and proteostasis under endoplasmic reticulum stress, both of which have been implicated in myopic sclera (Zhu et al, 2020). By contrast, ribosomal down-regulation in the choroid aligns with its thinning in spontaneously myopic animals (Jiang et al, 2022) and with reports of choroidal atrophy in pathologic myopia (Ohno-Matsui et al, 2016). Similar ribosomal alterations have been reported in defocus-induced chick myopia retina (Riddell et al, 2018), suggesting that translational regulation is a conserved hallmark of myopic eye growth across models (Metlapally & Wildsoet, 2015).

ECM–receptor interaction and focal adhesion pathways also showed a clear retina-to-sclera transition, with transcriptional down-regulation in the retina but proteomic up-regulation in the choroid and sclera. These pathways coordinate cell adhesion, matrix turnover, and mechanotransduction, and their protein-level enrichment likely reflects the implementation of matrix remodeling during ocular elongation. In parallel, contractility-related signaling was also altered in spontaneous myopia. Down-regulation of a contractility-associated pathway annotated as hypertrophic cardiomyopathy in the choroid in AM versus AH may indicate attenuated contractile or cytoskeletal tension during progressive remodeling (Bianco et al, 2024). Together with the choroidal thinning observed in spontaneously myopic animals (Jiang et al, 2022), this finding is consistent with altered biomechanical and vascular regulation during ocular elongation (Zhou et al, 2021). The consistent up-regulation of developmental regulators such as ZIC3 and BTC across tissues further suggests persistence of a conserved pro-growth program despite tissue-specific divergence during spontaneous-myopia pathogenesis.

Beyond contractile and structural remodeling, spontaneously myopic eyes were marked by broad metabolic reprogramming (Hou et al, 2023). Pathways including complement and coagulation cascades, fatty acid metabolism, glycolysis/gluconeogenesis, pentose phosphate pathway, proteasome, and purine metabolism were consistently down-regulated across the choroid and sclera. Selective up-regulation of proteins such as NFYA and SPCS1 in certain tissue pairs suggests stabilization of key transcriptional and translational regulators amid global metabolic suppression. Together, these changes are consistent with a shift toward an energy-conserving (Lin et al, 2024b), matrix-remodeling phenotype (Harper & Summers, 2015), in which resources are redirected from biosynthesis toward tissue adaptation during ocular elongation.

It is worth noting that regulators such as ZIC3, BTC, NFYA, and RRN3 were recurrently identified across tissues. Although this does not imply direct cross-tissue coordination, it suggests that a limited set of transcriptional and translational nodes may be associated with molecular changes in more than one ocular compartment in the spontaneous-myopia state.

Integrating the multi-tissue findings suggests that the transition from susceptibility-associated to spontaneously myopic state involves a gradual molecular shift rather than an abrupt change. In contrast to the near-complete retinal pathway switch observed in induced myopia models (Tkatchenko et al, 2018), the retina in spontaneous myopia showed only subtle, sustained transcriptional changes, suggesting that intrinsic signaling rather than visual feedback predominates. The choroid and sclera maintained overlapping pathways across both susceptibility and spontaneous myopia—including focal adhesion, complement and coagulation cascades, glycolysis/gluconeogenesis, and the pentose phosphate pathway—implying continuous engagement of structural and metabolic remodeling mechanisms. The reciprocal regulation of glycolysis/gluconeogenesis and the pentose phosphate pathway may reflect metabolic reprogramming toward rapid ATP generation at the expense of biosynthetic and antioxidant capacity (Yu et al, 2020), consistent with the energetic demands of ECM remodeling and vascular adaptation (Lin et al, 2024a; Baksh et al, 2024). Together, these data indicate that in the susceptibility-associated AH state, shared choroidal–scleral programs may support later elongation through coordinated metabolic, vascular, and structural remodeling.

Overall, these findings support a two-phase model of spontaneous myopia: retinal transcriptional changes may contribute to a permissive environment for growth, whereas the choroid and sclera show molecular features associated with the biomechanical and structural remodeling linked to axial elongation. Unlike induced paradigms, spontaneous myopia may represent a more intrinsically regulated, tissue-coupled progression. In line with human data, myopic eyes exhibit reduced responsiveness to growth-inhibitory cues in the choroid when subjected to plus-lens treatment (Swiatczak & Schaeffel, 2022), further suggesting that spontaneous elongation is shaped by inherent growth programs as well as external visual influences.

Comparison with human GWAS data revealed a similar overall pattern: risk loci were broadly represented in both choroid and sclera among susceptibility-associated genes, but were concentrated in the sclera among myopia-related genes. This supports a model in which genetic susceptibility primes both choroidal and scleral tissues, whereas scleral remodeling becomes more prominent during structural progression.

This interpretation is consistent with large-scale GWAS showing that refractive error is genetically heterogeneous and influenced by genes expressed across multiple ocular tissues (Hysi et al, 2020). Several established myopia-associated genes, including *CNTN4, CYP26A1, KCNA4, LRRC4C, PAX6*, and *RASGRF1*, were detected in both myopia-susceptible and spontaneously myopic GPs. Although many of these loci are best known for roles in neurodevelopment and synaptic regulation (Nishina et al, 1999; Fernandez-Medarde et al, 2009; Choi et al, 2019; Bamford et al, 2024), their expression in the choroid and sclera, however, implicates ECM organization, fibroblast-related remodeling, retinoic acid signaling, and mechanotransduction in ocular growth (Abu-Abed et al, 2001; Lobler et al, 2013; Uhlen et al, 2015). This is in line with GWAS and transcriptomic studies linking neuronal development and ECM pathways to myopia susceptibility and progression (Kiefer et al, 2013; Palumaa et al, 2024).

CYP26A1 provides a representative example of this multi-level regulation. In our data, *CYP26A1* mRNA was increased in the retina and choroid in the susceptibility and/or spontaneous-myopia comparisons, broadly consistent with reports of elevated retinal and choroidal retinoic acid in experimental myopia (McFadden et al, 2004). However, CYP26A1 protein did not increase correspondingly: semi-quantitative Western blot showed only a non-significant upward trend in the choroid but a significant decrease in the sclera. This transcript-protein discordance is compatible with post-transcriptional regulation, altered protein stability, or rapid turnover. In this context, AH should not be viewed as a simple linear intermediate between PH and AM. Rather, AH may represent a distinct susceptibility-associated state that remains phenotypically hyperopic although already showing RA-related regulatory alterations, whereas transition to AM may involve additional reprogramming or loss of compensatory regulation rather than uniform continuation of early changes. Thus, opposite directionality in some GWAS-linked genes does not argue against susceptibility but instead supports the view that predisposition and progression represent biologically distinct phases.

More broadly, *CYP26A1*/CYP26A1 illustrates how GWAS-linked susceptibility may first be reflected at the transcriptional level, whereas the sclera exhibits a distinct protein-level pattern more consistent with remodeling-related execution. Further studies of CYP26 enzymatic activity, RA metabolites, and cell-type-specific expression will be needed to distinguish among altered protein stability, reduced enzymatic function, and increased RA synthesis as the dominant mechanism. Integration of bulk multi-omics with single-cell or spatial transcriptomic approaches will be needed to define the cell-type localization of *CYP26A1*/CYP26A1, particularly in choroid and sclera, where the present data indicate transcript-protein discordance. Recent single-cell work in the guinea pig retina highlights the feasibility of this direction (Chen et al, 2025), although equivalent resources for the posterior wall remain limited.

These findings suggest a two-stage framework in which GWAS-prioritized genes may contribute first to a priming phase involving the retina and choroid and later to a more sclera-centered remodeling phase. Defining the regulatory checkpoints that separate early priming from later structural execution may help identify stage-specific intervention windows, such as targeting retinal or retinoid signaling early to limit initiation, or modulating scleral matrix remodeling to slow progression once myopia is established.

This study has several limitations that should be considered when interpreting the findings. First, transcriptomic and proteomic analyses were conducted in separate animal cohorts with limited sample sizes, which may have introduced individual variability, reduced power to detect gene–protein concordance or discordance, and precluded assessment of sex-specific molecular effects. Second, tissue dissection and bulk-layer analysis may have masked region-specific differences, particularly given the spatially restricted mRNA clustering observed in the retina, whereas RNAscope validation was exploratory, performed on a small number of samples without biological replicates, and therefore provides qualitative rather than definitive quantitative confirmation. Third, transcriptomic and proteomic profiles were assessed at a single developmental time point, and validation experiments were performed in slightly older animals (5 versus 2 wk), limiting direct interpretation of temporal relationships and introducing potential developmental differences in pathway activity. Finally, although several key genes and pathways were identified, functional studies will be required to establish causality and determine whether these molecular changes directly drive myopia susceptibility and spontaneous myopia.

Despite these limitations, the integration of transcriptomic and proteomic data across multiple ocular coats provided a powerful systems-level view of myopia development, increasing confidence in the biological relevance of the findings. Taken together, these findings provide an integrated cross-tissue framework for understanding molecular states associated with myopia susceptibility and spontaneous myopia. They highlight candidate pathways for future mechanistic investigation and support the value of spontaneous myopia as a complementary model for studying biological processes relevant to myopia susceptibility and progression.

## Materials and Methods

### Experimental design

The primary aim of this study was to explore molecular signaling networks associated with eye growth and their links to myopia susceptibility and spontaneous myopia through a multi-omics approach. Two GP strains were included: albino animals, which exhibit either spontaneous myopia or hyperopia and represent a myopia-prone strain with an inherited background that may contribute to the greater susceptibility to myopia induction observed in prior work, and pigmented GPs with hyperopia, which served as a myopia-resistant strain.

Both male and female animals were included in this study. For omics analyses, samples were grouped according to refractive phenotype, and sex-stratified analyses were not performed because of the limited sample size within each group.

**Figure 10.**
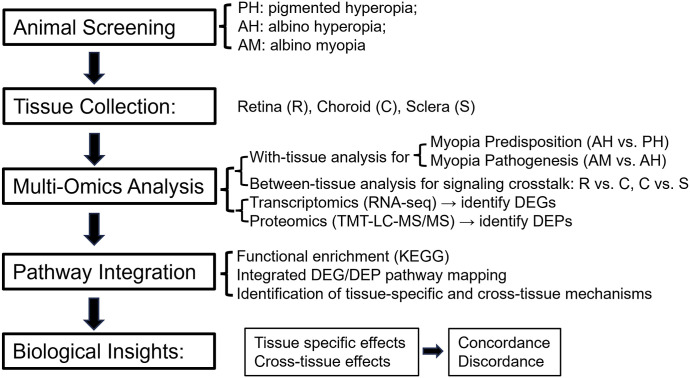
Experimental design. Comparison within the same tissue and between different refractive groups. DEGs, differentially expressed genes; DEPs, differentially expressed proteins; AH, albino hyperopic GPs; PH, pigmented hyperopic GPs; AM, albino myopic GPs.

Retina, choroid, and sclera tissues were dissected for transcriptomic and proteomic analyses. The comparison of AH versus PH was used to identify molecular signatures associated with susceptibility, whereas the comparison of AM versus AH was used to explore the molecular features associated with spontaneous myopia. The overall experimental design is summarized in Fig 10.

### Animals and housing

Albino and pigmented GPs (Elm Hill Labs) were maintained on a 12-h light/12-h dark cycle with ad libitum access to water and food and were bred on site. Breeding pairs were restricted to unrelated animals, and pups remained with their parents until P14. All animal care and experimental procedures adhered to the ARVO Statement for the Use of Animals in Ophthalmic and Vision Research. Protocols were approved by the Institutional Animal Care and Use Committee of SingHealth (AAALAC Accredited, 2021/SHS/1640).

### Refractive error screening and grouping

Refractive error was initially assessed at postnatal day 7. Based on the bimodal distribution observed in our previous study (Jiang et al, 2011), representative albino pups with typical myopia or hyperopia and pigmented pups with hyperopia were selected for follow-up. Cycloplegic refraction was confirmed at P14, and only animals showing consistent bilateral myopia or hyperopia at both time points were included.

Cycloplegic refraction was measured in awake animals using streak retinoscopy 30 min after topical instillation of 1% cyclopentolate hydrochloride (Bausch & Lomb) in the morning. Data are reported as spherical equivalents (SE) with a precision of ±0.25 D. Axial length (AL) was measured in awake animals using A-scan ultrasonography (10 MHz PacScan 300A; Sonomed Escalon) after topical corneal anesthesia with 0.5% Alcaine eye drops (Alcon).

Three experimental groups were established: albino myopic (AM, n = 9; SE: −4.00 D to −10.00 D), albino hyperopic (AH, n = 9; SE: +3.00 D to +8.00 D), and pigmented hyperopic (PH, n = 9; SE: +3.00 D to +8.00 D). For each animal, one eye was used for transcriptomic analysis (n = 4 per group) or proteomic analysis (n = 5 per group), and the other eye was reserved for other procedures.

### Tissue dissection

All tissue dissections were performed in the early afternoon (1:00–4:00 pm), and freshly enucleated eyes were kept on ice throughout the procedure. The anterior segment was removed via a perilimbal incision, followed by gentle removal of the lens and vitreous. The retina was carefully scraped and collected into 1.5 ml tubes. The eyecup was then radially cut and flattened, and the choroid (with residual RPE) was gently separated from the sclera and collected using fine tweezers. The remaining scleral tissue was cut into smaller pieces and collected. All tissues were flash-frozen in liquid nitrogen. Proteomic samples were stored at −80°C. For transcriptomic analysis, RNAProtect (QIAGEN) was added before storage at 4°C (short-term, ≤4 wk) or −20°C (long-term) until further processing.

### Transcriptomic analysis

Tissues were homogenized in lysis buffer using a TissueLyzer II bead homogenizer (QIAGEN) at 4°C with one 5-mm stainless steel bead per sample. Tissues were washed with Buffer RPE (QIAGEN), and RNA was extracted using the RNeasy Plus Mini Kit (QIAGEN) for retina and choroid or the RNeasy Fibrous Tissue Mini Kit (QIAGEN) for sclera. RNA purity was assessed by 260/280 nm absorbance ratios (2.11–2.13) on a NanoDrop spectrophotometer (Thermo Fisher Scientific), and RNA integrity was assessed by RNA Integrity Number (RIN = 9.0–10.0) using the RNA Nano 6000 kit on the Agilent 2100 Bioanalyzer (Agilent).

Sequencing libraries were prepared using the TruSeq Stranded mRNA Kit (Illumina), with 300 ng of total RNA as input for most samples and 170 ng for one sample, both within the manufacturer’s recommended range. Library fragment size was then determined by the Agilent 2100 Bioanalyzer using the DNA1000 kit. Libraries were sequenced on the Illumina HiSeq4000 System with 150-bp paired-end reads, generating ∼60–65 million read pairs per sample, sufficient to detect high, moderate, and low-expression genes.

Raw sequencing quality was assessed using FastQC v0.11.9 (http://www.bioinformatics.babraham.ac.uk/projects/fastqc). Adapter trimming was performed with Trimmomatic v0.36, (Bolger et al, 2014) followed by a second FastQC check to confirm complete adapter removal. Trimmed reads were aligned to the *Cavia porcellus* reference genome (CavPor3.0, ENSEMBL 100 [Howe et al, 2021]) using STAR v2.7.5+ (Dobin et al, 2013). Gene counts were generated with htseq-count v0.13.5 (Putri et al, 2022). The raw and processed sequencing data are available in the NCBI Gene Expression Omnibus (GEO) under accession number GSE256200.

For limma analyses, normalized log counts were modeled using a linear model with design = ∼batch + condition. Empirical Bayes moderation was applied using default parameters, and contrasts were specified to compare AH versus PH and AM versus AH within each tissue. Differential expression analysis was performed using limma with empirical Bayes moderation (Gentleman et al, 2004; Love et al, 2014; Huber et al, 2015). Genes with average counts ≤5 were excluded. DEGs were identified primarily using adjusted *P*-values for formal reporting, whereas nominal *P*-values and fold changes were used for visualization and rank-based downstream analyses. The full limma analysis script is provided as Supplemental Data 1 to facilitate reproducibility.

Supplemental Data 1. R Scripts.

### Proteomic analysis

Tissue lysis and protein extraction were performed using the EasyPep Mini MS Sample Prep Kit (Thermo Fisher Scientific) according to the manufacturer’s instructions. Protein concentration was determined using the DC Protein Assay (Bio-Rad Laboratories Inc.). Extracted peptides were reconstituted in kit assay solution, and peptide concentration was quantified with the Pierce Quantitative Fluorescent Peptide Assay (Thermo Fisher Scientific).

Peptides were labeled after the TMTpro 16plex Label Reagent Set protocol (Thermo Fisher Scientific). Aliquots of the peptide solutions from 15 samples of each tissue (retina, choroid, and sclera) were pooled and fractionated into eight fractions using the Pierce High pH Reversed-Phase Peptide Fractionation Kit (Thermo Fisher Scientific). Fractions were reconstituted in 0.1% formic acid and 5% acetonitrile, then analyzed on an EASY-nLC 1200 system coupled to an Orbitrap Exploris 480 mass spectrometer (Thermo Fisher Scientific). Liquid chromatography–tandem mass spectrometry (LC–MS/MS) data were processed in Proteome Discoverer v2.5 (Thermo Fisher Scientific) and searched against the Guinea pig Uniprot Reference Proteome (32,019 proteins, computationally analyzed). The mass spectrometry proteomics data have been deposited to the ProteomeXchange Consortium via the PRIDE (Perez-Riverol et al, 2025) partner repository with the dataset identifier PXD069847.

Given the limited sample size and lower sensitivity of proteomic measurements relative to RNA sequencing, nominal *P*-values (*P* < 0.05) combined with a more stringent fold-change threshold (|log_2_FC| > 0.75) were used to identify DEPs for integrative analyses.

PCA was conducted after applying a variance-stabilizing transformation from the DEP package to correct for dispersion and normalization factors. PCA plots were generated using the *prcomp* function in R v4.1.2 and customized with ggplot2 v3.1.1 (Wickham, 2016).

To assess tissue-specific and cross-tissue patterns, overlap analyses were performed for up-regulated and down-regulated genes and proteins, as well as between transcriptomic and proteomic layers. Because of variable numbers of differentially expressed biomolecules, we applied adaptive thresholds for selection. For within-layer comparisons, the top 1% of genes or proteins were selected based on a combined score calculated as log_2_ (fold change) × –log_10_ (*P*-value).

### Pathway enrichment analysis

Pathway enrichment analyses were performed using rank-based Gene Set Enrichment Analysis (GSEA v3.0), thereby avoiding reliance on arbitrary significance cutoffs and enabling detection of coordinated but modest changes across gene and protein sets.

Biological pathways were sourced from the Kyoto Encyclopedia of Genes and Genomes (KEGG; c2.cp.kegg.v7.2.symbols.gmt) via the Molecular Signatures Database (MSigDB; https://www.gsea-msigdb.org/gsea/msigdb). Unless otherwise noted, pathways with an adjusted enrichment *P*-value ≤ 0.05 were considered significant.

### Integration with published myopia genetics

To place our findings in the context of human myopia genetics, we cross-referenced DEGs with loci reported in GWAS. A reference list of myopia-associated genes was compiled from three comprehensive reviews (Cai et al, 2019; Tedja et al, 2019; Wang et al, 2022), supplemented by GWAS published in PubMed between 2020 and 2022 (Hysi et al, 2020; Tideman et al, 2021; Fuse et al, 2022). This integrative framework enabled us to identify molecular overlaps between our multi-omics datasets and established human myopia loci, thereby strengthening the translational relevance of our findings and their potential applicability to human myopia research.

### RNAscope in situ hybridization

RNAscope in situ hybridization was performed using the RNAscope 2.5 HD Duplex Detection Kit (Advanced Cell Diagnostics) according to the manufacturer’s instructions. Briefly, paraffin-embedded eye sections were deparaffinized, subjected to target retrieval and protease treatment, and hybridized with the CYP26A1 probe (red, catalog number: 1327281-C2). Signal detection was carried out using the chromogenic duplex system provided in the kit. Slides were counterstained with hematoxylin, air-dried, and mounted with a permanent mounting medium before imaging.

### RNAscope image analysis

RNAscope images were acquired using a Nikon Eclipse microscope (Nikon Corporation) at 20×, 40×, and 100× magnification. Images used for puncta counting were analyzed primarily at 40×, with 100× examination used where necessary to resolve closely clustered signals. Images were opened in Fiji (ImageJ, NIH), and spatial calibration was applied using microscope metadata to set the scale in μm/pixel. For each section, the entire visible area of each tissue layer, retina, choroid, and sclera, was manually outlined using the Freehand Selection tool. These whole-layer outlines were used to define the measurement areas for puncta counting, rather than selecting smaller subregions within each tissue. This procedure was repeated for sections from both the superior and inferior regions of the posterior eye. The outlined tissue-layer areas were measured (μm^2^) using the Measure function. CYP26A1 RNAscope puncta were counted manually by visual inspection across the entire outlined area of each tissue layer in a blinded manner. For each tissue layer, dot counts were normalized to the corresponding measured tissue-layer area and expressed as puncta per 100,000 μm^2^.

### Western blotting

Total protein was extracted from tissues using RIPA lysis buffer (R0010-100; BIO-RAD Laboratories). Protein concentrations were determined in duplicate using the BCA Protein Assay Kit (23227; Thermo Fisher Scientific). Equal amounts of protein (30 μg per lane) were resolved on 10% SDS–polyacrylamide gels and transferred to polyvinylidene fluoride (PVDF) membranes (88518; Thermo Fisher Scientific).

After transfer, total protein on the PVDF membranes was labeled using the No-Stain Protein Labeling Reagent (A44449; Thermo Fisher Scientific) according to the manufacturer’s instructions and imaged for total protein normalization. Membranes were then blocked with 5% skim milk and incubated overnight at 4°C with primary antibody against CYP26A1 (MBS9140790, 1:1,000; MyBioSource). After three washes with TBST, membranes were incubated with horseradish peroxidase-conjugated anti-rabbit IgG secondary antibody (211-032-171, 1:10,000; Jackson ImmunoResearch) for 1 h at room temperature. Immunoreactive bands were detected using chemiluminescence (SignalFire Plus ECL Reagent, #12630; Cell Signaling Technology) and imaged with a ChemiDoc MP Imaging System (BIO-RAD Laboratories). All images used for densitometric analysis were acquired under non-saturated exposure conditions.

Band densitometry was performed using ImageJ (NIH). CYP26A1 band intensity was normalized to the corresponding total protein signal from the same lane on the same membrane. Total protein was quantified as the integrated density of each sample lane within the detectable molecular weight range, excluding background. Data are presented as relative fold change in normalized CYP26A1 expression from three biological replicates.

### Statistical analysis

Refractive error and axial length at P14 in the animals pre-selected for omics profiling were analyzed using one-way ANOVA across AH, AM, and PH groups, followed by Tukey’s multiple-comparisons test. Western blot densitometric data, after normalization to the corresponding total protein signal, were analyzed using the same approach. Data are presented as mean ± SD unless otherwise indicated. As the omics cohort was pre-selected on the basis of refractive phenotype, the analysis shown in Fig 1 was intended to document the phenotypic characteristics of the selected animals, rather than to provide an unbiased estimate of group differences in the source population. Western blot results were derived from three independent biological replicates per group and are, therefore, presented as supportive evidence, to be interpreted with appropriate caution. Statistical significance was defined as *P* < 0.05.

## Supplementary Material

Reviewer comments

## Data Availability

The raw and processed sequencing data generated in this study have been deposited in the NCBI Gene Expression Omnibus (GEO) with the link https://www.ncbi.nlm.nih.gov/geo/query/acc.cgi?acc=GSE256200. The mass spectrometry proteomics data have been deposited to the ProteomeXchange Consortium via the PRIDE partner repository with the dataset identifier PXD069847. All other relevant data supporting the findings of this study are available within the article and its supplementary files.
