# Supplementary material for: Cross-tissue dual-omics analysis reveals molecular programs linked to myopia susceptibility and progression
Source: Life Sci Alliance. 2026 Jun 29;9(9):e202503595. doi: 10.26508/lsa.202503595 (PMC13315480; doi:10.26508/lsa.202503595)
Supplement: Supplementary file 4 [file LSA-2025-03595_TableS4.docx]

Supplementary Table S4. Statistical metrics for cross-tissue and cross-omics pathway enrichment in AH versus PH

| **AH vs PH** | **Retina** | | **Choroid** | | **Sclera** | |
| --- | --- | --- | --- | --- | --- | --- |
| **Enrichment pathways** | **mRNA** | **Protein** | **mRNA** | **Protein** | **mRNA** | **Protein** |
| **Oxidative phosphorylation** |  | **NES = 2.21**  **FDR = 0.006** |  | **NES = -1.80**  **FDR = 0.03** | **NES = -2.15**  **FDR < 0.001** | **NES = -2.10**  **FDR = 0.01** |
| **Cardiac muscle contraction** |  | **NES = 2.16**  **FDR = 0.002** | **NES = -1.88**  **FDR < 0.001** |  | **NES = -1.74**  **FDR = 0.02** |  |
| **Ribosome** |  |  | **NES = 2.07**  **FDR = 0.002** | **NES = 2.76**  **FDR < 0.001** | **NES = -2.60**  **FDR < 0.001** |  |
| **Antigen processing and presentation** |  |  |  | **NES = -1.86**  **FDR = 0.02** | **NES = -1.98**  **FDR = 0.002** |  |
| **Arginine and proline metabolism** |  |  |  |  | **NES = -1.71**  **FDR = 0.02** | **NES = -2.23**  **FDR = 0.003** |
| **Complement and coagulation cascades** |  |  |  |  | **NES = -1.75**  **FDR = 0.02** | **NES = 1.72**  **FDR = 0.005** |
| **Drug metabolism cytochrome P450** |  |  |  |  | **NES = -1.88**  **FDR = 0.005** | **NES = -1.96**  **FDR = 0.02** |
| **ECM receptor interaction** | **NES = -1.83**  **P = 0.001** | **NES = 1.94**  **FDR = 0.03** |  |  |  |  |
| **Glutathione metabolism** |  |  |  |  | **NES = -1.83**  **FDR = 0.01** | **NES = -2.05**  **FDR = 0.02** |
| **Glycolysis gluconeogenesis** |  |  |  | **NES = -2.29**  **FDR < 0.001** |  | **NES = -2.50**  **FDR < 0.001** |
| **Leukocyte transendothelial migration** |  |  |  | **NES = 1.72**  **FDR < 0.05** | **NES = -1.84**  **FDR = 0.009** |  |
| **Lysosome** |  |  |  | **NES = -2.29**  **FDR < 0.001** | **NES = -1.66**  **FDR = 0.03** |  |
| **Metabolism of xenobiotics by cytochrome P450** |  |  |  |  | **NES = -2.02**  **FDR = 0.002** | **NES = -1.91**  **FDR = 0.02** |
| **Neuroactive ligand receptor interaction** |  |  | **NES = -1.75**  **FDR = 0.006** |  | **NES = 2.10**  **FDR < 0.001** |  |
| **Regulation of actine cytoskeleton** |  |  |  | **NES = 2.00**  **FDR = 0.007** |  | **NES = 1.54**  **FDR = 0.23** |
| **Tight junction** |  |  |  | **NES = 1.74**  **FDR < 0.05** | **NES = -1.62**  **FDR = 0.04** |  |
| **Vascular smooth muscle contraction** |  |  |  | **NES = 2.37**  **FDR < 0.001** |  | **NES = 1.95**  **FDR = 0.01** |
| **Alanine aspartate and glutamate metabolism** |  |  |  | **NES = -1.69**  **FDR < 0.05** |  |  |
| **Calcium signaling pathway** |  |  | **NES = -1.51**  **P < 0.001** |  |  |  |
| **Cell adhesion molecules** |  |  |  |  | **NES = -1.68**  **FDR = 0.03** |  |
| **Cysteine and methionine metabolism** |  |  |  | **NES = -1.74**  **FDR = 0.04** |  |  |
| **Cytokine-cytokine receptor interaction** |  |  |  |  | **NES = -1.71**  **FDR = 0.02** |  |
| **Cytosolic DNA sensing pathway** |  |  |  |  | **NES = -1.73**  **FDR = 0.02** |  |
| **Dilated cardiomyopathy** |  |  | **NES = -1.62**  **FDR = 0.08** |  |  |  |
| **Focal adhesion** |  |  |  | **NES = 1.50**  **P < 0.001** |  |  |
| **Fructose and mannose metabolism** |  |  |  | **NES = -1.77**  **FDR = 0.04** |  |  |
| **Galactose metabolism** |  |  |  | **NES = -1.74**  **FDR = 0.04** |  |  |
| **Hematopoietic cell lineage** |  |  |  |  | **NES = -2.09**  **FDR < 0.001** |  |
| **Hypertrophic cardiomyopathy** |  |  | **NES = -1.60**  **FDR = 0.07** |  |  |  |
| **Intestinal immune network for IGA production** |  |  |  |  | **NES = -1.79**  **FDR = 0.01** |  |
| **Leishmania infection** |  |  |  |  | **NES = -1.74**  **FDR = 0.02** |  |
| **Long term depression** |  |  |  |  | **NES = 1.65**  **P < 0.01** |  |
| **Mapk signaling pathway** |  |  |  |  |  | **NES = 1.67**  **P < 0.001** |
| **Natural killer cell mediated cytotoxicity** |  |  |  |  | **NES = -1.94**  **FDR = 0.003** |  |
| **Olfactory transduction** |  |  |  | **NES = -1.73**  **FDR = 0.04** |  |  |
| **Pentose phosphate pathway** |  |  |  |  |  | **NES = -1.93**  **FDR = 0.02** |
| **peroxisome** |  |  |  |  |  | **NES = -1.88**  **FDR = 0.02** |
| **Phosphatidylinositol signaling system** |  |  |  |  |  | **NES = 1.89**  **FDR = 0.01** |
| **PPAR signaling pathway** |  |  |  |  |  | **NES = -1.93**  **FDR = 0.02** |
| **Primary immunodeficiency** |  |  |  |  | **NES = -1.99**  **FDR = 0.002** |  |
| **Propanoate metabolism** |  |  |  |  |  | **NES = -1.99**  **FDR = 0.02** |
| **proteasome** |  |  |  |  | **NES = -2.06**  **FDR = 0.001** |  |
| **Protein export** |  |  |  | **NES = 1.87**  **FDR = 0.02** |  |  |
| **spliceosome** |  | **NES = -1.83**  **P < 0.001** |  |  |  |  |
| **Starch and sucrose metabolism** |  |  |  |  |  | **NES = -1.84**  **FDR = 0.03** |
| **T-cell receptor signaling pathway** |  |  |  |  | **NES = -1.61**  **FDR = 0.04** |  |
| **Tryptophan metabolism** |  |  |  |  | **NES = -1.79**  **FDR = 0.01** |  |
| **Viral myocarditis** |  |  |  |  | **NES = -2.20**  **FDR < 0.001** |  |
