# Supplementary material for: Cross-tissue dual-omics analysis reveals molecular programs linked to myopia susceptibility and progression
Source: Life Sci Alliance. 2026 Jun 29;9(9):e202503595. doi: 10.26508/lsa.202503595 (PMC13315480; doi:10.26508/lsa.202503595)
Supplement: Supplementary file 8 [file LSA-2025-03595_TableS8.docx]

Supplementary Table S8, Statistical metrics for cross-tissue and cross-omics pathway enrichment in AM versus AH

| **AM vs AH** | **Retina** | | **Choroid** | | **Sclera** | |
| --- | --- | --- | --- | --- | --- | --- |
| **Enrichment pathways** | **mRNA** | **Protein** | **mRNA** | **Protein** | **mRNA** | **Protein** |
| **Ribosome** | **NES = -1.94**  **FDR = 0.03** | **NES = 1.73**  **P = 0.006** | **NES = -2.19**  **FDR = 0.001** |  | **NES = 2.33**  **FDR = 0.00** | **NES = 2.23**  **FDR < 0.001** |
| **ECM receptor interaction** | **NES = -1.80**  **FDR < 0.05** |  |  | **NES = 2.44**  **FDR = 0.00** |  | **NES = 2.23**  **FDR < 0.001** |
| **Focal adhesion** | **NES = -1.75**  **FDR = 0.08** |  |  | **NES = 1.98**  **FDR = 0.001** |  | **NES = 1.41**  **FDR = 0.1** |
| **Hypertrophic cardiomyopathy** | **NES = -1.73**  **FDR = 0.07** |  |  | **NES = 2.05**  **FDR < 0.001** |  | **NES = -1.70**  **FDR = 0.03** |
| **Cardiac muscle contraction** |  |  |  | **NES = 2.12**  **FDR < 0.001** |  | **NES = -1.97**  **FDR = 0.004** |
| **Complement and coagulation cascades** |  |  |  | **NES = -2.53**  **FDR < 0.001** |  | **NES = -2.41**  **FDR < 0.001** |
| **Fatty acid metabolism** |  |  |  | **NES = -2.08**  **FDR = 0.002** |  | **NES = -1.70**  **FDR = 0.03** |
| **Glycolysis gluconeogenesis** |  |  |  | **NES = -1.92**  **FDR = 0.01** |  | **NES = -2.08**  **FDR < 0.001** |
| **Pentose phosphate pathway** |  |  |  | **NES = -2.36**  **FDR < 0.001** |  | **NES = -1.62**  **FDR < 0.05** |
| **proteasome** |  |  |  | **NES = -1.86**  **FDR = 0.01** |  | **NES = -1.84**  **FDR = 0.007** |
| **Purine metabolism** |  |  |  | **NES = -1.66**  **FDR = 0.06** |  | **NES = -1.65**  **FDR < 0.05** |
| **Viral myocarditis** | **NES = -1.90**  **FDR = 0.01** |  |  |  |  | **NES = -1.65**  **FDR = 0.04** |
| **Apoptosis** |  |  |  |  |  | **NES = -1.62**  **FDR = 0.05** |
| **Arginine and proline metabolism** |  |  |  |  |  | **NES = -1.76**  **FDR = 0.02** |
| **B-cell receptor signaling pathway** |  |  |  |  |  | **NES = -1.62**  **FDR = 0.05** |
| **Calcium signaling pathway** |  |  |  |  |  | **NES = -1.77**  **FDR = 0.02** |
| **Dilated cardiomyopathy** |  |  |  | **NES = 2.00**  **FDR < 0.001** |  |  |
| **Epithelial cell signaling in helicobacter pylori infection** |  |  |  |  |  | **NES = -1.58**  **FDR = 0.06** |
| **Fructose and mannose metabolism** |  |  |  | **NES = -2.05**  **FDR = 0.004** |  |  |
| **Leukocyte transendothelial migration** |  |  |  |  |  | **NES = -1.62**  **FDR = 0.05** |
| **Long term depression** |  |  |  |  |  | **NES = -1.62**  **FDR < 0.05** |
| **Neuroactive ligand receptor interaction** |  |  |  |  | **NES = -1.79**  **FDR = 0.01** |  |
| **Oxidative phosphorylation** |  |  |  |  |  | **NES = -1.90**  **FDR = 0.005** |
| **Prion diseases** |  |  |  | **NES = -1.86**  **FDR = 0.01** |  |  |
| **Pyruvate metabolism** |  |  |  | **NES = -1.90**  **FDR = 0.01** |  |  |
| **Small cell lung cancer** |  |  |  | **NES = 2.01**  **FDR < 0.001** |  |  |
| **spliceosome** |  |  |  |  |  | **NES = 1.93**  **FDR = 0.006** |
| **Starch and sucrose metabolism** |  |  |  |  |  | **NES = -1.74**  **FDR = 0.02** |
| **Tight junction** |  |  |  |  |  | **NES = -1.74**  **FDR = 0.02** |
| **Tryptophan metabolism** |  |  |  | **NES = -2.01**  **FDR = 0.004** |  |  |
| **Vascular smooth muscle contraction** | **NES = -1.92**  **FDR = 0.02** |  |  |  |  |  |
