# Supplementary material for: Cross-tissue dual-omics analysis reveals molecular programs linked to myopia susceptibility and progression
Source: Life Sci Alliance. 2026 Jun 29;9(9):e202503595. doi: 10.26508/lsa.202503595 (PMC13315480; doi:10.26508/lsa.202503595)
Supplement: Reviewer comments [file LSA-2025-03595_review_history.pdf]

Cross-tissue dual-omics analysis reveals molecular programs linked to myopia susceptibility and progression

Liqin Jiang, Lijin Wang, Lei Zhou, Fangquan Wu, Johnny Ma, Willie Yu, James H.Z. Koh, Audrey Chia, Seang-Mei Saw, Sujoy Ghosh, and Veluchamy Barathi
DOI: https://doi.org/10.26508/lsa.202503595

Corresponding author(s): Veluchamy Barathi, Singapore Eye Research Institute and Sujoy Ghosh, Pennington Biomedical Research Center

Review Timeline:

Submission Date:	2025-12-12
Editorial Decision:	2026-02-24
Revision Received:	2026-05-02
Editorial Decision:	2026-05-27
Revision Received:	2026-05-31
Accepted:	2026-06-10

Scientific Editor: Sarita Hebbar

Transaction Report:

(Note: With the exception of the correction of typographical or spelling errors that could be a source of ambiguity, letters and reports are not edited. The original formatting of letters and referee reports may not be reflected in this compilation.)

February 25, 2026

Re: Life Science Alliance manuscript #LSA-2025-03595-T

Dr. Liqin Jiang
Singapore Eye Research Institute
Translational Pre-Clinical Model Platform
Singapore

Dear Dr. Jiang,

Thank you for submitting your manuscript entitled "Dual-omics of spontaneous myopia reveals a primed state and conserved ribosomal signal" to Life Science Alliance. Your manuscript was assessed by three experts whose comments are appended below.

As you will note all the reviewers are consistent in their conclusion that your study provides a significant advance in the area of myopia pathogenesis. These reviewers have also raised several concerns, which we agree, preclude publication at this stage. We draw your attention to the concern of Reviewer 2 on RNAscope images (points 7-8) and agree that you must provide a clear explanation on these data. Overall, we invite you to submit a revised manuscript addressing all of the reviewers' concerns on presentation of these results, and the general description of this work and its discussion.

When submitting the revision, please include a letter addressing the reviewers' comments point by point. While a rebuttal must respond to all points in some form, additional experiments to resolve these points are not required.

The typical timeframe for revisions is three months. Please note that papers are generally considered through only one revision cycle, so strong support from the referees on the revised version is needed for acceptance.

I would be happy to discuss the revision in more detail via email or phone/videoconferencing. Please let me know which option you prefer, if any.

To upload the revised version of your manuscript, please log in to your account: https://lsa.msubmit.net/cgi-bin/main.plex

You will be guided to complete the submission of your revised manuscript and to fill in all necessary information. Please get in touch in case you do not know or remember your login name.

Thank you for this interesting contribution to Life Science Alliance. We hope that the comments below will prove constructive as your work progresses, and we are looking forward to receiving your revised manuscript.

Sincerely,

Sarita Hebbar, PhD
Scientific Editor
Life Science Alliance
http://www.lsajournal.org

A. THESE ITEMS ARE REQUIRED FOR REVISIONS

-- A letter addressing the reviewers' comments point by point.

-- An editable version of the final text (.DOC or .DOCX) is needed for copyediting (no PDFs).

-- High-resolution figure, supplementary figure and video files uploaded as individual files: See our detailed guidelines for preparing your production-ready images, https://www.life-science-alliance.org/authors

-- Summary blurb (enter in submission system): A short text summarizing in a single sentence the study (max. 200 characters including spaces). This text is used in conjunction with the titles of papers, hence should be informative and complementary to the title and running title. It should describe the context and significance of the findings for a general readership; it should be written in the present tense and refer to the work in the third person. Author names should not be mentioned.

-- By submitting a revision, you attest that you are aware of our payment policies found here: https://www.life-science-alliance.org/copyright-license-fee

B. MANUSCRIPT ORGANIZATION AND FORMATTING:

Full guidelines are available on our Instructions for Authors page, https://www.life-science-alliance.org/authors

We encourage our authors to provide original source data, particularly uncropped/-processed electrophoretic blots and spreadsheets for the main figures of the manuscript. If you would like to add source data, we would welcome one PDF/Excel-file per figure for this information. These files will be linked as supplementary "Source Data" files.

IMPORTANT: It is Life Science Alliance policy that if requested, original data images must be made available. Failure to provide original images upon request will result in unavoidable delays in publication. Please ensure that you have access to all original microscopy and blot data images before submitting your revision.

Reviewer #1 (Comments to the Authors (Required)):

Jiang L, et al., presented a manuscript characterizing the transcriptomics and proteomics in the myopia-prone and myopia-resistant guinea pig models. They investigated three types of guinea pig models, including albino spontaneous myopia, albino hyperopia, and pigmented hyperopia, and examined three ocular tissues, the retina, choroid, and sclera. The authors reported a two-stage molecular model based on their dual-omics analysis: a predisposition phase, which is characterized by widespread transcript-protein discordance in the choroid and sclera, and a pathogenesis phase, which is featured by active tissue remodeling with concordant transcript-protein expression. Additionally, they specified the ribosome/translation pathway as a consistent signal among tissues examined and validated the expression of CYP26A1. This study provides new insights into the molecular and cellular signaling pathways underlying myopia development, and the manuscript is well written. However, minor revisions are needed before it can be considered for publication.

Major Questions:
1. Introduction
1) Page 4, Line 58, 59: please revise the myopia prevalence. The cited figures (23% for myopia and 2.7% for high myopia) reflect data from 2000; however, these rates have increased significantly. The projected myopia prevalence from Holden's paper: 2020 is ~34% and 2030 ~40%.
2) Page 4, Line 70: Add references for "experimentally induced models"
3) Page 4, Line 70-71: Add references for "spontaneous models of myopia"
4) Page 4, Line 73-77: This paragraph should be merged with the last paragraph of Introduction (Page 5 Line 86-92)
2. Results (Page 6 line 132): Molecular predisposition to myopia development (AH vs. PH)
1) The overall rationale for comparing AH vs PH at Day 14 is that differential gene and protein expression at this time determines their susceptibility to myopia induction. Is it possible that it is not the case? Alternatively, baseline differences may not dictate the response to induction; rather, the divergent patterns of eye growth may result from distinct physiological responses during the induction phase itself.
2) Page 7 Line 155-Page 8 Line 175, same as AM vs AH results: Another primary rationale for data analysis is to identify the "overlapping genes and proteins" across two or three tissues. While signaling pathways from the retina to the choroid and then sclera may occur sequentially, is it logically sound to identify the same molecules in different tissues simultaneously?
3) Page 7, Line 152: I could not find the "Supplementary Table 1a". "Supplementary Table1s" (1-1 to 1-6) (Page7 Line 147) is the only Supplementary tables provided.
3. Discussion
1) Page 16 Line 373 - Page 18 Line 412: please revise the whole section. It is hard to follow.
For example, Line 377 discussed sclera, Line 379 continue to discuss sclera or retina - not clear? Line 381 discuss sclera again? Then this paragraph ended with "choroid" as a relay? Please revise to make it clear if this is a summary paragraph.
Next paragraph is retina, ...
Next paragraph jumped to concordant changes across layers? Interaction between choroid, retina and sclera?
Next paragraph is scleral again?
Next paragraph is choroid
4. Figure 1: If statistics is made between AH and AM, please indicate it clearly in the figure accordingly

Minor Questions:
1. Introduction (Page 4, Line 73-74): [8][9] should be [8,9]
2. Page 14 Line 324: typo for "Differential", should be "differential"
3. Format the references consistently

Reviewer #2 (Comments to the Authors (Required)):

In the work entitled "Dual-omics of spontaneous myopia reveals a primed state and conserved ribosomal signal," Jiang and colleagues took advantage of naturally occurring refractive phenotypes in albino guinea pigs, performing transcriptomic and proteomics comparisons between pigmented guinea pigs (PH), myopic albino guinea pigs (AM), and hyperopic albino (AH) guinea pigs. Analysis was performed on retinal, choroidal, and scleral tissues from all three groups, with emphasis on comparisons between PH and AH, AH and AM, and comparisons with overall findings from GWAS. Interestingly, despite a seemingly high degree of variation in transcriptional findings between groups, the change at the protein level is much smaller. While it has long been established that translation does not necessarily equate with translation, this study emphasizes this concept across multiple ocular tissues and provides a comparative approach to understanding differences that contribute to myopia susceptibility and the myopic disease state in wild-type animals. These findings provide an important perspective on interpreting transcriptional changes in myopic models, as scRNA sequencing is currently the predominant methodology used in the field. Based on this information, the interpretation of these studies may require further validation at the protein level. Ultimately, omics worked described provides value to the field of myopia research; however, there are some concerns relating to the validation studies using RNAscope and western blotting techniques that require addressing. Please see a list of concerns below:
Conclusions:
Together, these results suggest that while transcriptional regulation shows broad cross-tissue commonalities, proteomic overlap is more restricted, indicating that baseline susceptibility to myopia may be established by shared genetic programs but refined by tissue-specific protein regulation.

1. Overall, the figures could benefit from more explicit labeling and improved organization to enhance readability.
2. The authors should disclose whether both sexes were used and if so, whether there was any sex-dependent clustering.
3. The authors refer to "warm" and "cold" colors in the figure legends. They should just say red or blue.
4. In Figure 1, the authors report using a Student's t-test. The authors should verify the correct use of statistical tests. Since three groups are being compared, the authors should use a one-way ANOVA.
5. In Figure1, the authors show bar graphs. These graphs should be displayed in a format that allows readers to see individual values rather than solid bars.
6. In figure 8, the authors use RNAscope to evaluate the expression of Cyp26a1 in the retina choroid and sclera. The authors show OCT images, but never actually talk about OCT or reference those images in the text. Furthermore, there are two images shown for each group. The authors do not distinguish between the top and bottom image.
7. Figure 8 continued, in the figure legends the authors state that the images are "qualitative and not intended for definitive quantification" but then go on to quantify this assay in table 5. The authors should provide clarification here.
a. Was quantification of RNAscope images performed? If yes, was it on these images. If not these, then which images?
b. The images shown do not show RNAscope performed at a resolution that allows for quantification. Several of the images show groupings that are too close together to distinguish individual dots for counting.
c. If the authors did quantify RNAscope images, they should include this as a graph with Figure 8 and provide information related to how many samples were evaluated (animal number, sections, ROIs, etc.).
d. The authors should annotate the images to help distinguish retina, choroid, and sclera.
8. Figure 9 shows western blot analysis with whole protein as the loading control. Despite indicating that the same amount of protein was loaded into each well, it appears that there is significantly more total protein in the PH samples. The authors should address this
9. The authors should address melatonin and its potential influence any comparisons between PH and AH.
10. In line 564 it appears that there was an editorial comment left in the text "(exactly what pathways)." I agree that these pathways should be described here.
11. Finally, I do not feel like the title does not match the overall conclusions of the manuscript.

Reviewer #3 (Comments to the Authors (Required)):

This study by Jiang et al is a valuable approach to better understand the molecular mechanism of myopia. The transcriptional and proteomic analyses are thoroughly performed and provide novel insights into the potential molecular mechanisms of development of myopia. However, the interpretation of the model and some details of the differential expression require further consideration, as outlined below.

1. The meaning of concordance and discordance, particularly as used in the abstract, should be more explicitly explained (ie RNA and protein expression changing in the same direction).

2. Regulation of gene expression is very complicated, occurring at many levels (transcription, RNA stability, mRNA export, translation efficiency, protein stability etc) Further discussion of this process is required to accompany interpretation of the findings. For example, how does concordance between RNA and protein indicate active remodeling? Discuss the need for a time course experiment to better understand lack of concordance between mRNA and protein levels.

3. Evaluation of both protein and RNA expression is a strength of the study. Explain in more detail how this enables distinction of

molecular signatures of susceptibility from those of progression. It would be helpful to include an explanation of how discordance between mRNA and protein changes should be interpreted in the Introduction.

4. Comparison of spontaneously myopic albinos versus their hyperopic littermates is justified to detect differences due to the condition. The comparisons between albino and pigmented animals are described as identifying 'baseline differences linked to susceptibility'. The authors should explain why these differences are linked exclusively to myopia susceptibility rather than presence or absence of pigment? For example, PMEL was unsurprisingly the most consistently differentially expressed between albino and pigmented guinea pigs, but is this gene directly linked to potential development of myopia? I may have misunderstood the design, but pigmented hyperopic were compared with albino hyperopic guinea pigs - if the albinos were hyperopic does this mean that they are the ones that didn't actually spontaneously develop myopia, or are they still at risk of developing myopia if left to live longer?

5. Although the samples clustered by tissue, it is an over-simplification to state that 'all samples within each tissue clustered tightly, with no outliers detected' (Line 129). There is a greater spread within choroid and sclera samples, which although well separated from each other by marker gene expression, do come close to each other on the PCA plot. Indeed, the spread observed in PC1 is likely due to the extent of contamination with retina tissue (this is apparent on a PCA plot coloured by rhodopsin expression - available on request).
Nonetheless, these appear to be high quality samples and this variation does not compromise the analyses, but is probably worth noting. The authors should also assess whether the greater dispersion of choroid and sclera samples might contribute to the discovery of more DEGs than in retina when using low stringency cut-offs for FC and p value.

6. More caution required when comparing transcriptomic and proteomic differential expression. The 'striking transcript-protein discrepancy' may be more due in large part to the different sensitivities/biases of the two approaches. More justification required to state that 'large-scale transcriptional priming occurs in the choroid and sclera' (Line 149).

7. Clarify which parameters were used in which analyses and justify use of nominal values and small absolute change of 1.2 (ie 20% increase):
Transcriptomic analysis. Methods Line 641' Differentially expressed genes were filtered by adjusted p-value and log2 fold-change'
Proteomic. Line 662: 'Proteins with Ilog2 (fold change)I > 0.75 and p-value < 0.05 were considered differentially expressed'.
Results Line 143 'Using a threshold of absolute fold-change {greater than or equal to} 1.2 and nominal p {less than or equal to} 0.05, transcriptomic changes were considerably more extensive than proteomic ones.'

8. Provide further justification for the statement that 'these results suggest that while transcriptional regulation shows broad cross-tissue commonalities, proteomic overlap is more restricted' (Line 171). When taking into account the fewer DEPs, is the overlap really significantly less? For example, of 54 DEGs 7 are shared, of 33 DEPs 3 are shared - both close to a proportion of 0.1.

9. Comment on why 'a larger set of pathways showed strong concordance' line 209.

10. Comparison with GWAS is a powerful approach. It is striking that all changes in GWAS genes differentially expressed in 'AH vs PH' and 'AM vs AH' change in the opposite direction (Fig 7). For these genes, this suggests that PH is more similar to AM than AH - is this consistent with AH being a susceptible state?

11. There are many changes in expression to discuss, but it would be helpful, if possible, to condense this the Discussion section.

12. Suggestion for additional work: Look for cell type expression of CYP26A1 in single cell data (mouse?)
Eg consider and cite:
Minyu Chen, Jiahui Liu, Guimei Liang, Qingyang Liu, Shuyan Li, Yifang Yang.Cross-species and cross-platform analysis reveals the application value of guinea pig retina in myopia research at single-cell resolution. Experimental Eye Research, Volume 259, 2025, 110558, ISSN 0014-4835. https://doi.org/10.1016/j.exer.2025.110558.

• Minor corrections and suggestions:
Explain what aspect of CYP26A1 is being 'validated' (in Abstract).
Justify quantification of chemiluminescence signal from western blots. Band densitometry semi quantitative at best.
Perhaps an Upset plot might be suitable to illustrate overlap between DEGs/DEPs in the different tissues.
Reword 'In total, 24, 30, and 19 genes were commonly upregulated in the retina-choroid, choroid-sclera, and retina-sclera pairs, respectively' (Line 159). Suggest 'In total, 24 genes were upregulated in retina-choroid, 30 in choroid-sclera, and 19 in retina-sclera comparisons.'
PCA figure - colour in legend to match symbols (red and orange!)
Include GP = Guinea Pig in abbreviations
Line 623: 260/280 nm absorbance ratios (2.11-2.13) on a Nanodrop (Thermo Fisher Scientific) does not measure RNA integrity -

it quantifies and assesses purity.
Provide more details of default values used for limma, ideally provide script as supplementary. Facilitate replication.
Line 414 During the myopia pathogenesis stage, most pathways showed concordant regulation. - clarify that referring to concordance between changes in the three tissues.

Dear Editor,

We sincerely thank you and Reviewers for your careful evaluation of our manuscript and for the constructive and insightful comments. We appreciate the time and effort invested in improving the quality and clarity of our work.

We have carefully addressed all comments, and our point-by-point responses are outlined below. We have made additional editing changes to improve clarity. We feel that all comments were insightful and greatly improve the manuscript.

We hope that the revisions adequately address the concerns raised and that the manuscript is now suitable for publication in Life Science Alliance.

Dear Dr. Jiang,

Thank you for submitting your manuscript entitled "Dual-omics of spontaneous myopia reveals a primed state and conserved ribosomal signal" to Life Science Alliance. Your manuscript was assessed by three experts whose comments are appended below.

As you will note all the reviewers are consistent in their conclusion that your study provides a significant advance in the area of myopia pathogenesis. These reviewers have also raised several concerns, which we agree, preclude publication at this stage. We draw your attention to the concern of Reviewer 2 on RNAscope images (points 7-8) and agree that you must provide a clear explanation on these data. Overall, we invite you to submit a revised manuscript addressing all of the reviewers' concerns on presentation of these results, and the general description of this work and its discussion.

When submitting the revision, please include a letter addressing the reviewers' comments point by point. While a rebuttal must respond to all points in some form, additional experiments to resolve these points are not required.

The typical timeframe for revisions is three months. Please note that papers are generally considered through only one revision cycle, so strong support from the referees on the revised version is needed for acceptance.

I would be happy to discuss the revision in more detail via email or phone/videoconferencing. Please let me know which option you prefer, if any.

To upload the revised version of your manuscript, please log in to your account: https://ddei5-0-ctp.trendmicro.com:443/wis/clicktime/v1/query?url=https%3a%2f%2flsa.msubmit.net%2fcgi%2dbin%2fmain.plex&umid=832466EF-4BA2-1306-B7C8-960AE62546E4&auth=d4a022de4fa71b6d10c50609f2626ca32dd9d36f-4330518457cddb55575ec9f1af2eb97b8a16197f

You will be guided to complete the submission of your revised manuscript and to fill in all necessary information. Please get in touch in case you do not know or remember your login name.

Thank you for this interesting contribution to Life Science Alliance. We hope that the comments below will prove constructive as your work progresses, and we are looking forward to receiving your revised manuscript.

Sincerely,

Sarita Hebbar, PhD

Scientific Editor

Life Science Alliance

Response:

Thank you, Dr. Hebbar. We appreciate your guidance here. We have confirmed that all the required information is provided in the revised manuscript

Response to Reviewer #1 – General Comment

Jiang L, et al., presented a manuscript characterizing the transcriptomics and proteomics in the myopia-prone and myopia-resistant guinea pig models. They investigated three types of guinea pig models, including albino spontaneous myopia, albino hyperopia, and pigmented hyperopia, and examined three ocular tissues, the retina, choroid, and sclera. The authors reported a two-stage molecular model based on their dual-omics analysis: a predisposition phase, which is characterized by widespread transcript-protein discordance in the choroid and sclera, and a pathogenesis phase, which is featured by active tissue remodelling with concordant transcript-protein expression. Additionally, they specified the ribosome/translation pathway as a consistent signal among tissues examined and validated the expression of CYP26A1. This study provides new insights into the molecular and cellular signalling pathways underlying myopia development, and the manuscript is well written. However, minor revisions are needed before it can be considered for publication.

Response:

We thank the Reviewer for the positive and thoughtful summary of our work.

We have carefully addressed all comments raised below and have further refined the manuscript to improve clarity, strengthen mechanistic interpretation, and enhance the presentation of the dual-omics integration across ocular tissues.

Major Questions:
1.1. Introduction
1.1.1) Page 4, Line 58, 59: please revise the myopia prevalence. The cited figures (23% for myopia and 2.7% for high myopia) reflect data from 2000; however, these rates have increased significantly. The projected myopia prevalence from Holden's paper: 2020 is ~34% and 2030 ~40%.

Response:

Thank you for this important update. We agree that more recent prevalence estimates better reflect the current and projected global burden of myopia. We have revised this section to incorporate updated projections based on Holden et al., with additional supporting references, indicating a prevalence of ~34% in 2020 and ~40% by 2030.

Revision made:

Page 4, lines 67-70, as follow:

"*Global myopia prevalence is projected to increase from approximately 23% in 2000 to nearly 50% by 2050 (Holden BA et al, 2016). Based on this model, subsequent analyses estimate that prevalence reached approximately 34% in 2020 and is expected to approach 40% by 2030 (Nemeth J et al, 2021; Pan W et al, 2025).*"

1.1.2) Page 4, Line 70: Add references for "experimentally induced models"

Response:

We thank the Reviewer for this suggestion. Appropriate references describing experimentally induced models of myopia in different animal models have now been added.

Revision made:

Page 4, lines 85-86, as follow:

"*Most mechanistic knowledge comes from experimentally induced models (Hung LF et al, 1995; Norton TT et al, 2006; Schaeffel F et al, 1988; Wildsoet C & Wallman J, 1995).*"

1.1.3) Page 4, Line 70-71: Add references for "spontaneous models of myopia"

Response:

We thank the Reviewer for this suggestion. Appropriate references describing spontaneous models of myopia have now been added.

Revision made:

Page 4, lines 86-87, as follow:

"*Although spontaneous models of myopia are available (Jiang L et al, 2022; Jiang L et al, 2011), they remain underexplored despite their potential…*"

1.1.4) Page 4, Line 73-77: This paragraph should be merged with the last paragraph of Introduction (Page 5 Line 86-92)

Response:

We thank the reviewer for this helpful suggestion. We have substantially revised and integrated the final two paragraphs of the Introduction to improve the overall flow and to clarify the rationale, study design, and key conceptual framework of the study.

Revision made:

Page 5, lines 117-130, as follow:

"*Here, we implemented an integrated multi-omics approach to profile transcriptomic and proteomic signatures across the retina, choroid, and sclera in albino hyperopic (AH), albino spontaneously myopic (AM), and pigmented hyperopic (PH) guinea pigs (GP). Comparison of AH and PH animals, which share a hyperopic state but differ in pigmentation background and susceptibility, was used to identify molecular features associated with myopia predisposition. Comparison of AM and AH littermates was used to identify pathways associated with spontaneous myopia progression. By integrating transcriptomic and proteomic datasets, applying pathway enrichment analyses, and examining patterns of concordance and discordance across ocular tissues from the same eye, this dual-comparison design captures both susceptibility-related and myopia-associated states within the same species. Building on evidence*

for population-specific differences in myopia prevalence and progression (Hagen LA et al, 2018; Luong TQ et al, 2020), this framework improves biological interpretability and highlights molecular cascades with potential translational relevance."

1.2. Results (Page 6 line 132): Molecular predisposition to myopia development (AH vs. PH)
1.2.1) The overall rationale for comparing AH vs PH at Day 14 is that differential gene and protein expression at this time determines their susceptibility to myopia induction. Is it possible that it is not the case? Alternatively, baseline differences may not dictate the response to induction; rather, the divergent patterns of eye growth may result from distinct physiological responses during the induction phase itself.

Response:

We thank the reviewer for this important comment. We agree that the AH versus PH comparison at postnatal day 14 does not by itself establish that differential gene or protein expression at this stage directly determines later susceptibility to myopia induction. In this study, the concept of "susceptibility" is used as a biological framework grounded in prior animal work (Jiang et al., 2011, 2019) and as the rationale for comparing myopia-prone and myopia-resistant strains at a shared hyperopic stage. Our intention was therefore to identify early molecular profiles associated with susceptibility status, rather than to claim that the present data alone prove causality or exclude contributions from differential responses arising during the induction phase itself.

To address this concern, we have revised the manuscript throughout to distinguish more clearly between the study hypothesis and the direct inferences supported by the current cross-sectional multi-omics data. Specifically, we now use more descriptive language in the Results and have changed the subsection title.

Revision made:

Page 7, line 182, as follow:
"*Distinct molecular profiles in AH and PH despite a shared hyperopic state*"

We also note that some overlapping features in the AH vs PH comparison, such as PMEL, are likely to reflect pigmentation-related background differences rather than myopia susceptibility-specific mechanisms.

Page 8, lines 224-226, as follow:

"*Notably, some overlapping features in the AH vs PH comparison, such as PMEL, are likely to reflect pigmentation-related background differences rather than susceptibility-specific mechanisms."*

In addition, we clarify in the Discussion that the day 14 differences are interpreted as molecular features associated with susceptibility, while acknowledging that subsequent divergence in eye growth may also depend on physiological responses that emerge during the induction phase.

Page 17, lines 487-490, as follow:

"*The overall pattern suggests that susceptibility involves coordinated but tissue-specific molecular states across adjacent ocular layers. Subsequent divergence in eye growth, however, is likely to depend not only on these baseline molecular features but also on physiological responses that emerge during the induction phase.*"

1.2.2) Page 7 Line 155-Page 8 Line 175, same as AM vs AH results: Another primary rationale for data analysis is to identify the "overlapping genes and proteins" across two or three tissues. While signaling pathways from the retina to the choroid and then sclera may occur sequentially, is it logically sound to identify the same molecules in different tissues simultaneously?

Response:

We thank the reviewer for this insightful comment. We agree that the presence of the same molecules in multiple tissues at a single time point cannot be interpreted as direct evidence of either simultaneous or sequential signaling between those tissues. In the revised manuscript, we have clarified that the overlap analysis was intended as a descriptive strategy to identify shared or recurrent molecular features across ocular tissues, rather than to demonstrate directional propagation of specific signals from retina to choroid to sclera. We now interpret these overlapping genes and proteins as shared molecular signatures that may reflect common regulatory influences or parallel tissue responses across ocular compartments.

Revision made:

Page 8, lines 203-206, as follow:

"*We next compared overlaps among DEGs and DEPs across the three tissues using Venn analysis for up- and downregulated genes (Table 1a, Supplementary Table 2), as a descriptive strategy to identify shared or recurrent molecular features across ocular tissues rather than to infer direct inter-tissue signal propagation*."

Page 12, lines 318-319, as follow:

"*We next applied the same descriptive Venn-based strategy used for the AH vs PH comparison to the AM vs AH comparison. Analysis of the top 1% of DEGs (ranked by…*"

Our broader interest in cross-tissue signaling derives from the biological framework outlined in the Introduction and from the established concept that myopia-related remodeling involves multiple ocular compartments (Wallman and Winawer, 2004). The present overlap analysis alone cannot resolve the temporal order or mechanistic direction of those interactions. We have therefore revised the relevant text to replace stronger wording with more neutral terms, and we now present mechanistic interpretation as a working hypothesis rather than a direct conclusion from the overlap analysis.

1.2.3) Page 7, Line 152: I could not find the "Supplementary Table 1a". "Supplementary Table1s" (1-1 to 1-6) (Page7 Line 147) is the only Supplementary tables provided.

Response:

We thank the reviewer for bringing this to our attention. Supplementary Table 1a was inadvertently omitted from the supplementary files at submission. We have included it in the revised submission and have reviewed the numbering and cross-referencing of all supplementary tables in the manuscript to avoid further inconsistencies.

1.3. Discussion

1.3.1) Page 16 Line 373 - Page 18 Line 412: please revise the whole section. It is hard to follow.
For example, Line 377 discussed sclera, Line 379 continue to discuss sclera or retina - not clear? Line 381 discuss sclera again? Then this paragraph ended with "choroid" as a relay? Please revise to make it clear if this is a summary paragraph.
Next paragraph is retina, ...
Next paragraph jumped to concordant changes across layers? Interaction between choroid, retina and sclera?
Next paragraph is scleral again?
Next paragraph is choroid

Response:

We thank the reviewer for this careful reading and apologize that that the original Discussion section was not sufficiently clear in its sequence and organization. In response, we have substantially revised this section to improve logical flow, tissue-by-tissue structure, and readability.

Specifically, we reorganized the discussion to follow a clearer anatomical and conceptual sequence, moving from retina to choroid to sclera, so that tissue-specific interpretations are presented in a more coherent order.

In addition, we removed or softened wording that previously made the discussion appear to shift abruptly between tissues or between descriptive and mechanistic interpretation. Cross-tissue observations are now presented more clearly as integrative interpretation, rather than interspersed in a way that interrupted tissue-specific discussion. Overall, these revisions were made to improve clarity, internal logic, and alignment between paragraph structure and biological narrative.

Revision made:

Pages 16-17, lines 440-490, as follow:

"*Myopia susceptibility, as modeled here, was associated with coordinated changes in ECM-, immune- , and metabolism-related pathways across ocular tissues. Although*

the present data do not establish mechanism, these patterns may mark a molecular environment relevant to later ocular growth regulation.

In the retina, ECM-receptor interaction pathways showed marked transcript–protein discordance: broad transcriptional downregulation of collagens, laminins, and integrins contrasted with proteomic upregulation of selected laminin and proteoglycan components. Although the retina is predominantly neural, extracellular matrix and basement-membrane proteoglycan systems are well represented at retinal structural interfaces, supporting the relevance of ECM-associated pathway shifts in this tissue (Keenan TD et al, 2012). This pattern suggests that, despite reduced ECM-related transcripts, key structural proteins may be maintained through selective translation, differential turnover, or protein stabilization (Aisenbrey S et al, 2006). Such regulation may help preserve basement-membrane integrity and mechanotransductive responsiveness with the retina.

In parallel with the retinal findings, the choroid showed susceptibility-associated changes in neurochemical, redox, and contractile-associated pathways. Downregulation of the GABA transporters SLC6A11 and SLC6A1 indicates altered GABA-related signaling, consistent with previously reported strain-dependent differences in scotopic ERG responses (Jiang L et al, 2025). Reduced NEFL, TNNT3, and MYH1 may reflect diminished contractile or cytoskeletal responsiveness. Shared downregulation of GSTK1 additionally indicates increased oxidative vulnerability. Together, these changes support the view that the choroid may serve as an intermediate regulatory tissue in which neural-associated, metabolic, redox, and biomechanical influences converge before overt elongation (Tedja MS et al, 2018).

In the sclera, broad transcriptomic downregulation of immune-related pathways contrasted with proteomic upregulation of complement and coagulation cascades, again indicating marked transcript-protein discordance. Given prior evidence implicating cytokines and immune pathways in myopia development in both humans (Jiang L et al, 2024; Yuan J et al, 2019) and animal models (Lin HJ et al, 2016; Xiao K et al, 2025), one interpretation is that transcriptional immune quiescence may coexist with selective protein-level activation of complement-related pathways. Such a state could permit remodeling-related immune surveillance while limiting excessive inflammatory or cross-linking responses (Riddell N & Crewther SG, 2017; Zeng L et al, 2023), thereby favoring a more compliant scleral matrix, a biomechanical condition linked to axial elongation.

Consistent with these tissue-specific differences, the choroid and sclera showed complementary metabolic features, with ribosome upregulation in the choroid suggesting increased translational capacity, whereas downregulation of oxidative phosphorylation, arginine–proline metabolism, and cytochrome P450 pathways in the sclera indicated a relatively hypometabolic state.

Together, these findings support a cross-tissue framework in which retinal structural signaling, choroidal neurochemical and metabolic integration, and scleral immune-metabolic remodeling are each associated with myopia susceptibility. Although some differences are also likely to reflect pigmentation biology and broader strain-specific

background effects, circadian influences, including potential effects of melatonin signaling, cannot be fully excluded in the PH versus AH comparison, even though this risk was minimized by standardized housing conditions and a restricted tissue-collection window. The overall pattern suggests that susceptibility involves coordinated but tissue-specific molecular states across adjacent ocular layers. Subsequent divergence in eye growth, however, is likely to depend not only on these baseline molecular features but also on physiological responses that emerge during the induction phase."

1.4. Figure 1: If statistics is made between AH and AM, please indicate it clearly in the figure accordingly

Response:

We thank the reviewer for this important point. Because Figure 1 includes comparisons among three groups, ordinary one-way ANOVA followed by Tukey's multiple comparisons test is more appropriate than Student's t-test. We have therefore reanalyzed the data accordingly and revised Figure 1 to clearly indicate the relevant pairwise statistical comparisons across the AH, AM, and PH groups. The updated figure now includes significance brackets and annotations directly on the graphs, including the AH versus AM comparison.

We have also updated the Methods section to describe the statistical analysis used for Figure 1 (lines 846–857) and specified the test in the revised figure legend.

Revision made:

Pages 28-29, lines 852-863, as follow:

"*Statistical analysis*

Refractive error and axial length at P14 in the animals pre-selected for omics profiling were analyzed using one-way ANOVA across AH, AM, and PH groups, followed by Tukey's multiple-comparisons test. Western blot densitometric data, after normalization to the corresponding total protein signal, were analyzed using the same approach. Data are presented as mean ± SD unless otherwise indicated. As the omics cohort was pre-selected on the basis of refractive phenotype, the analysis shown in Figure 1 was intended to document the phenotypic characteristics of the selected animals, rather than to provide an unbiased estimate of group differences in the source population."

Figure 1 was updated to display pairwise comparisons across AH, AM, and PH, including AH versus AM, using significance brackets and annotations based on ordinary one-way ANOVA with Tukey's multiple comparisons test.

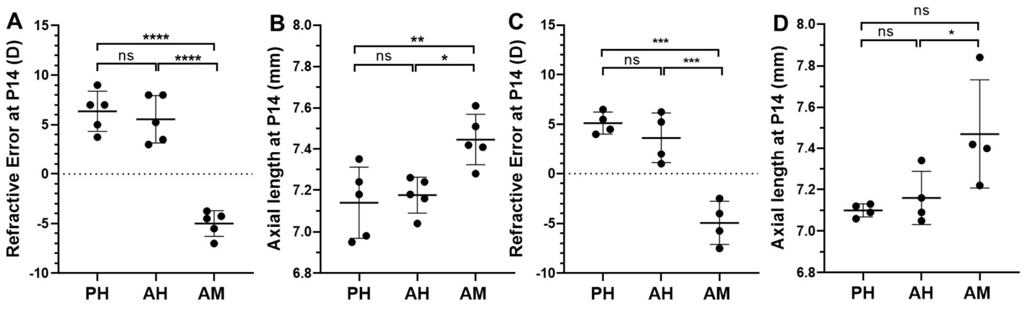

Figure legend: Page 37, lines 1170-1177, as follow:

"Figure 1. Refractive and axial phenotypes of the pre-selected omics cohort at postnatal day 14.

*(A, C) Cycloplegic refractive error and (B, D) axial length in AH, AM, and PH guinea pig (GP) selected for transcriptomic (A, B; n=4) and proteomic (C, D; n=5) analysis. Each symbol represents one animal; bars show mean ± SD. Ordinary one-way ANOVA with Tukey's multiple-comparisons test was used. Ns, Not Significant, *P <0.05; ***P<0.001; P****<0.0001. These comparisons are presented to confirm the phenotypic separation of the selected subset used for omics profiling."*

1.5. Minor Questions:
1.5.1. Introduction (Page 4, Line 73-74): [8][9] should be [8,9]

Response:

Thank you for the detail. It has been corrected.

1.5.2. Page 14 Line 324: typo for "Differential", should be "differential"

Response:

Thank you for the correction. This has now been resolved.

1.5.3. Format the references consistently

Response:

Thank you for this comment. We have downloaded the Life Science Alliance citation style in EndNote and systematically reformatted the references throughout the manuscript to ensure consistent formatting and compliance with the journal's author–year citation style.

Reviewer #2 (Comments to the Authors (Required)):

In the work entitled "Dual-omics of spontaneous myopia reveals a primed state

and conserved ribosomal signal," Jiang and colleagues took advantage of naturally occurring refractive phenotypes in albino guinea pigs, performing transcriptomic and proteomics comparisons between pigmented guinea pigs (PH), myopic albino guinea pigs (AM), and hyperopic albino (AH) guinea pigs. Analysis was performed on retinal, choroidal, and scleral tissues from all three groups, with emphasis on comparisons between PH and AH, AH and AM, and comparisons with overall findings from GWAS. Interestingly, despite a seemingly high degree of variation in transcriptional findings between groups, the change at the protein level is much smaller. While it has long been established that translation does not necessarily equate with translation, this study emphasizes this concept across multiple ocular tissues and provides a comparative approach to understanding differences that contribute to myopia susceptibility and the myopic disease state in wild-type animals. These findings provide an important perspective on interpreting transcriptional changes in myopic models, as scRNA sequencing is currently the predominant methodology used in the field. Based on this information, the interpretation of these studies may require further validation at the protein level. Ultimately, omics worked described provides value to the field of myopia research; however, there are some concerns relating to the validation studies using and western blotting techniques that require addressing. Please see a list of concerns below.
Conclusions: Together, these results suggest that while transcriptional regulation shows broad cross-tissue commonalities, proteomic overlap is more restricted, indicating that baseline susceptibility to myopia may be established by shared genetic programs but refined by tissue-specific protein regulation.

Response:

We thank the reviewer for this thoughtful and constructive overall assessment. We also appreciate the reviewer’s concerns regarding the RNAscope and Western blot validation studies. These concerns have been carefully addressed point-by-point below, and the manuscript has been revised accordingly.

2.1. Overall, the figures could benefit from more explicit labelling and improved organization to enhance readability.

Response:

We thank the reviewer for this important comment. In response, we revised Figures 1, 2, 3, 5, and 8 to improve readability through more explicit labeling and clearer overall organization.

Updated figures are as follows:

Figure 1.

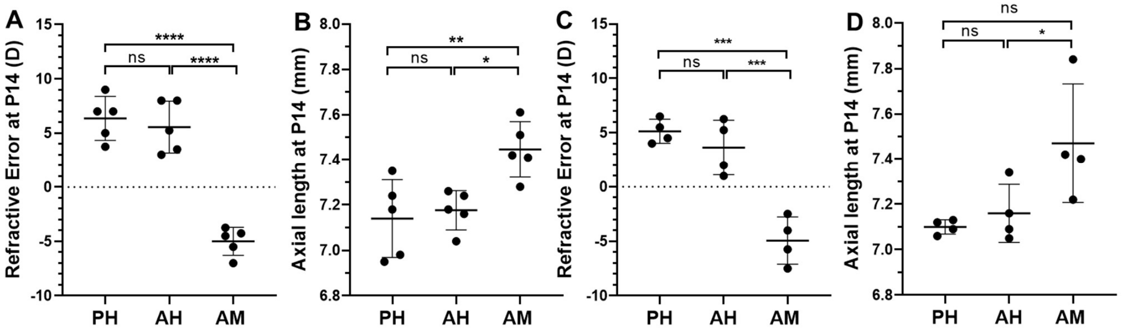
A
B
C
D
Refractive Error at P14 (D)
Axial length at P14 (mm)
PH
AH
AM
ns
*
**
***
****

Figure 2,

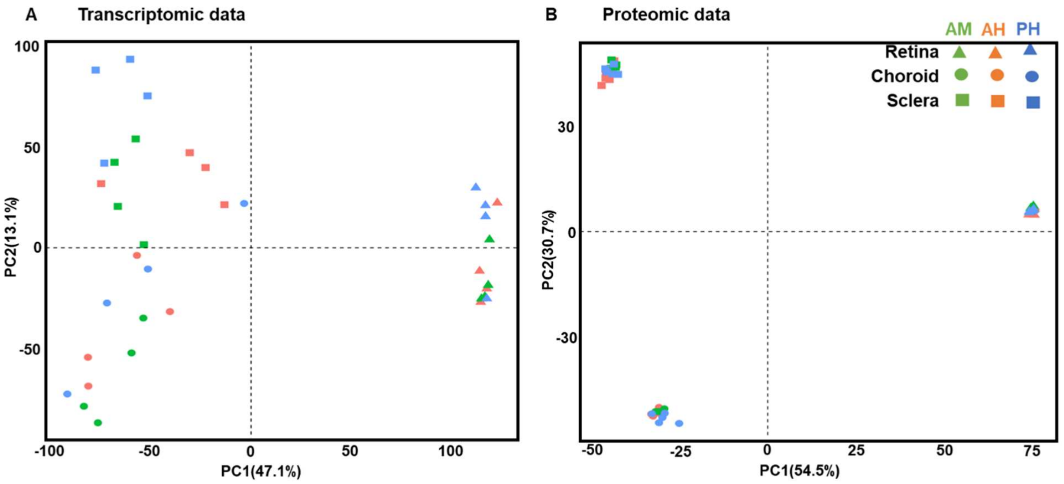
A
Transcriptomic data
B
Proteomic data
AM AH PH
Retina
Choroid
Sclera
PC1(47.1%)
PC2(13.1%)
PC1(54.5%)
PC2(30.7%)

Figure 3,

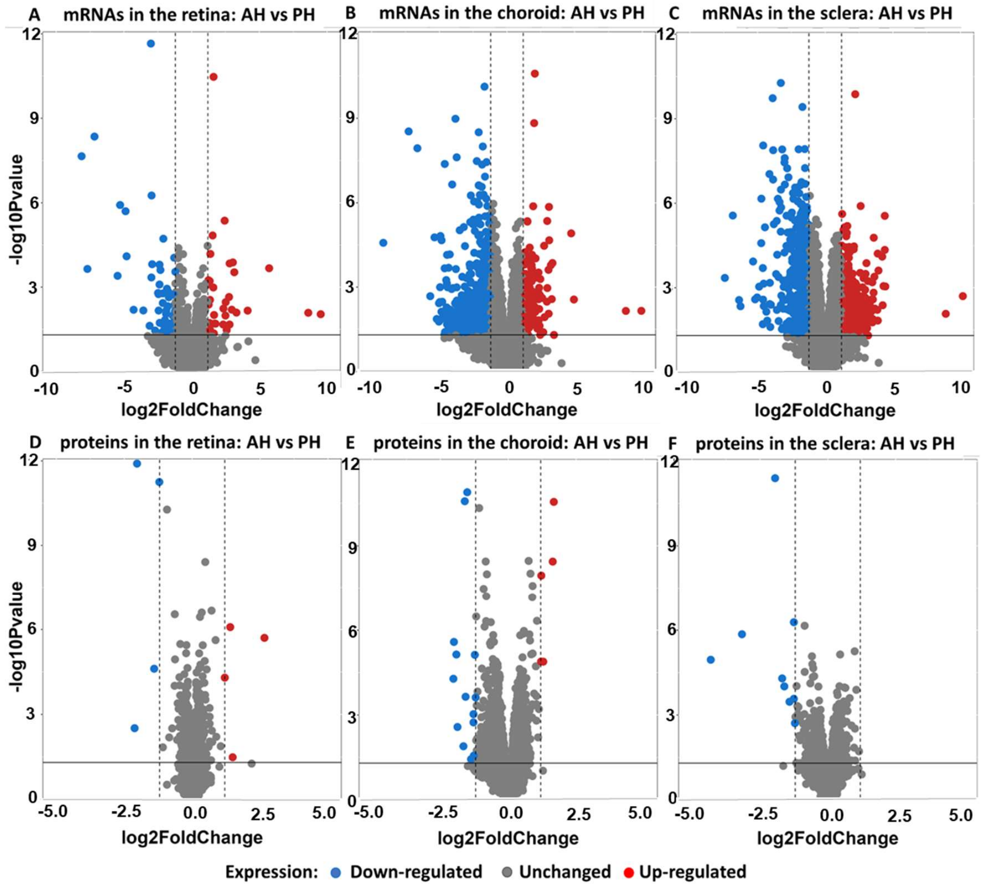
A mRNAs in the retina: AH vs PH
B mRNAs in the choroid: AH vs PH
C mRNAs in the sclera: AH vs PH
D proteins in the retina: AH vs PH
E proteins in the choroid: AH vs PH
F proteins in the sclera: AH vs PH
-log10Pvalue
log2FoldChange
Expression: Down-regulated Unchanged Up-regulated

Figure 5,

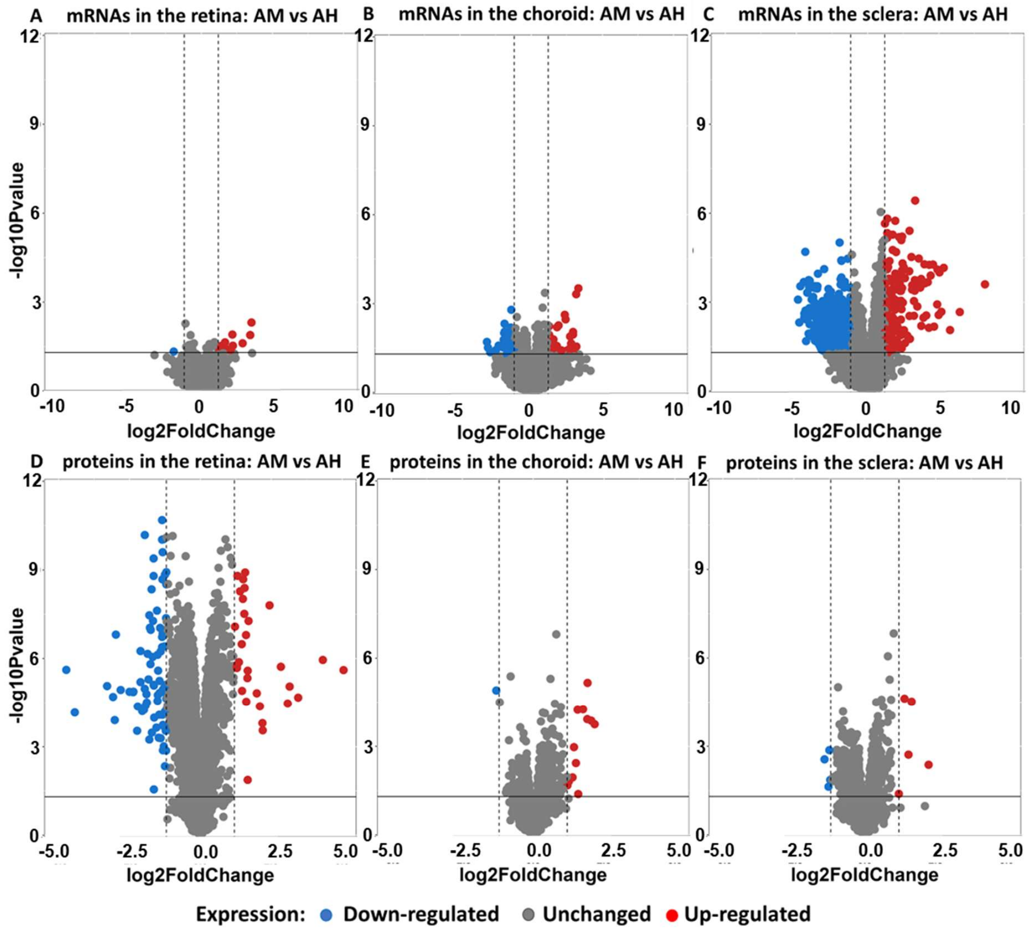

Figure 8,

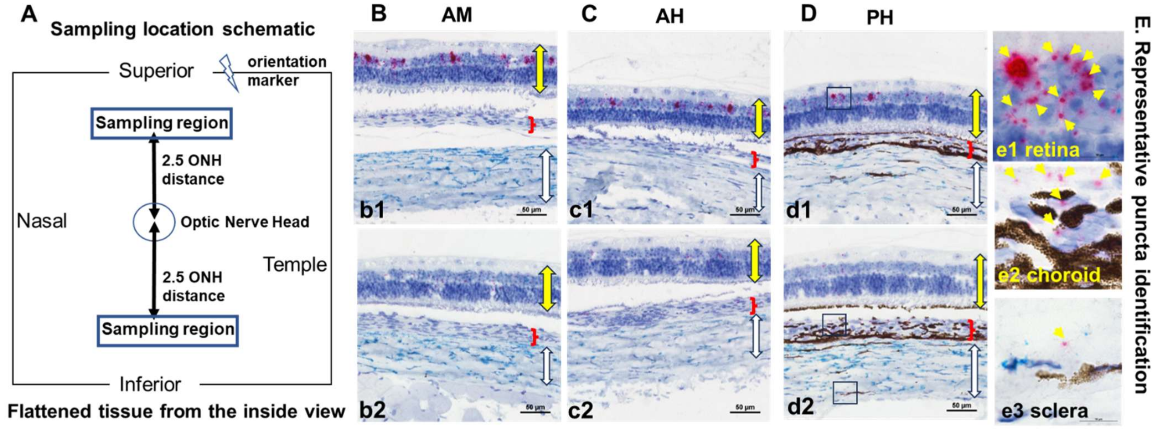

2.2. The authors should disclose whether both sexes were used and if so, whether there was any sex-dependent clustering.

Response:

We thank the reviewer for raising this important issue. Both male and female animals were included in this study. For the transcriptomic and proteomic analyses, samples were grouped based on refractive phenotype, as the aim of the present work was to identify molecular signatures associated with susceptibility and spontaneous myopia. Because the number of samples within each refraction-defined group was limited (transcriptomics, n = 4; proteomics, n = 5), the dataset was not adequately powered for sex-stratified clustering analysis. We therefore did not assess sex-dependent clustering and cannot draw robust conclusions regarding sex-specific effects from the current omics dataset. This has now been clarified in the revised Methods, and the lack of sex-stratified analysis is acknowledged as a limitation in the Discussion. We agree that sex is an important biological variable, and larger studies will be required to determine whether sex-dependent molecular patterns contribute to these phenotypes.

Revision made:

Page 23, lines 677-679, as follow:

"*Both male and female animals were included in this study. For omics analyses, samples were grouped according to refractive phenotype, and sex-stratified analyses were not performed because of the limited sample size within each group."*

Page 22, lines 625-628, as follow:

"*First, transcriptomic and proteomic analyses were conducted in separate animal cohorts with limited sample sizes, which may have introduced individual variability, reduced power to detect gene–protein concordance or discordance, and precluded assessment of sex-specific molecular effects*."

2.3. The authors refer to "warm" and "cold" colours in the figure legends. They should just say red or blue.

Response:

We thank the reviewer for the suggestion. The corresponding corrections have been made to the legends for Figures 4 and 6.

2.4. In Figure 1, the authors report using a Student's t-test. The authors should verify the correct use of statistical tests. Since three groups are being compared, the authors should use a one-way ANOVA.

Response:

We thank the reviewer for this important comment. We agree that one-way ANOVA is required because Figure 1 includes three groups: AH, AM, and PH. We have therefore reanalyzed the data using ordinary one-way ANOVA followed by Tukey's multiple comparisons test. Figure 1 has been revised to show the relevant pairwise comparisons with significance brackets and annotations, and the Methods section and figure legend have been updated accordingly. This revision has been described in our response to Comment 1.4 above.

2.5. In Figure1, the authors show bar graphs. These graphs should be displayed in a format that allows readers to see individual values rather than solid bars.

Response:

We thank the reviewer for this helpful suggestion. In response, Figure 1 has been revised to display the individual data points for each group rather than solid bar graphs alone, allowing the distribution of values within each group to be visualized more clearly. Mean ± SD has been retained to summarise the group-level data.

This revision is also described in our response to Comment 1.4 above.

2.6. In figure 8, the authors use RNAscope to evaluate the expression of Cyp26a1 in the retina choroid and sclera. The authors show OCT images, but never actually talk about OCT or reference those images in the text. Furthermore, there are two images shown for each group. The authors do not distinguish between the top and bottom image.

Response:

We thank the reviewer for this helpful observation. In the original Figure 8, the OCT images were included solely as a positional reference to indicate the approximate retinal regions used for histological sampling. They were not intended for quantitative or comparative analysis and were not acquired from the same animals used for RNAscope. We agree that the purpose of this panel was not sufficiently explained in the original figure and legend. In the revised manuscript, we have replaced the OCT images' panel with a **schematic diagram of the sampling locations** and have explicitly indicated the superior and inferior regions for the corresponding histological images shown with RNAscope staining.

Revision made:

Figure 8 was updated and legend was revised: page 38, lines 1215-1229, as follow:

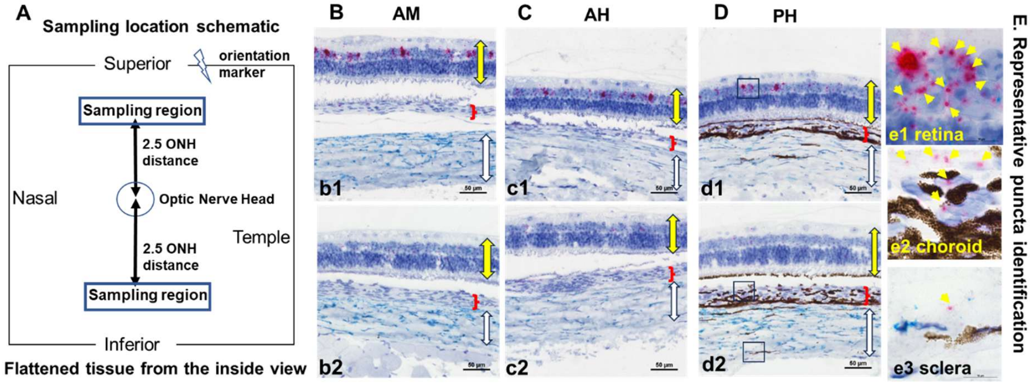

Figure 8. Regional distribution and exploratory assessment of condition-dependent CYP26A1 expressions. (A) Sampling scheme. In left eyes, sections for RNAscope were collected at approximately 2.5 optic nerve head (ONH) diameters from the ONH center in the superior and inferior regions. (B–D) Representative RNAscope images showing CYP26A1 mRNA (red puncta) in the retina (yellow double arrows), choroid (red brackets), and sclera (white double arrows) under spontaneous myopia (B, AM), myopia-prone (C, AH), and myopia-resistant (D, PH) states. For each group, the upper panel shows the superior region (b1, c1, and d1) and the lower panel shows the inferior region (b2, c2, and d2). (E) Representative examples of CYP26A1 RNAscope puncta identification in the retina (e1), choroid (e2), and sclera (e3). Boxed regions are shown at higher magnification (100x), and yellow arrows indicate counted puncta used for exploratory density measurement. n = 1 animal per group (5 weeks old). CYP26A1 signal was observed mainly in the retina, with lower signal in the choroid, and sparse signal in the sclera. Images are representative of the analyzed regions and are shown for illustrative purposes only. A corresponding exploratory semi quantitative assessment is provided in Table 5. Scale bars, 50 μm.

2.7. Figure 8 continued, in the figure legends the authors state that the images are "qualitative and not intended for definitive quantification" but then go on to quantify this assay in table 5. The authors should provide clarification here.
2.7.a. Was quantification of RNAscope images performed? If yes, was it on these images. If not these, then which images?

Response:

We thank the reviewer for this important clarification. Yes, RNAscope signal was assessed by image-based puncta-density measurement. Specifically, CYP26A1-positive puncta were counted and normalized to tissue area (puncta per 100,000 μm²). The measurements summarized in Table 5 were derived from the same stained sections from which the representative image fields shown in Figure 8 were selected. Thus, Figure 8 shows representative cropped regions from the analyzed sections, whereas Table 5 summarizes the corresponding exploratory puncta-density assessment. We agree that the original wording did not make this relationship sufficiently clear. We have therefore revised the relevant text accordingly and

updated the figure legend, as described in our response to Comment 2.6 above. The revised text is provided below.

Revision made in the text**:**

Pages 14-15, lines 401-404, as follow:

"*Representative RNAscope images (Figure 8) suggested region-dependent CYP26A1 signal, with stronger signal in the retina, lower signal in the RPE/choroid, and sparse signal in the sclera; corresponding exploratory puncta-density measurements from the analyzed sections are summarized in Table 5."*

2.7.b. The images shown do not show RNAscope performed at a resolution that allows for quantification. Several of the images show groupings that are too close together to distinguish individual dots for counting.

Response:

We thank the reviewer for this important observation. We agree that the representative panels shown in Figure 8 are not suitable for definitive puncta quantification by visual inspection alone. The displayed images were acquired at 20× and were included primarily to illustrate the regional distribution of CYP26A1 signal across the retina, choroid, and sclera. For image-based counting, however, we used higher-magnification images, specifically 40× for the retina and choroid and 100× for the sclera.

Regarding clustered signals in which individual puncta were difficult to distinguish, we carefully examined the higher-magnification images, particularly at 100×, and estimated puncta numbers as accurately as possible. Nevertheless, we agree that these measurements should be interpreted cautiously. Accordingly, the values presented in Table 5 are retained as an exploratory image-based assessment rather than as definitive quantification.

To improve clarity, we have also added a subset of magnified images as Figure 8E to better show the puncta at higher resolution, as described in our response to Comment 2.6 above. To avoid overstating these data, we have further revised the manuscript to clarify that the RNAscope analysis is exploratory in nature, and that the representative images are provided to illustrate regional signal distribution rather than to serve as stand-alone quantitative evidence.

2.7.c. If the authors did quantify RNAscope images, they should include this as a graph with Figure 8 and provide information related to how many samples were evaluated (animal number, sections, ROIs, etc.).

Response:

We thank the reviewer for this helpful suggestion. In the revised manuscript, we have added a graphical summary of the RNAscope puncta-density measurements to accompany Figure 8 and have expanded the figure legend and Methods text to

specify the basis of this assessment. Specifically, CYP26A1 signal was measured as puncta density per 100,000 µm² by manually counting puncta across the entire outlined area of each tissue layer — retina, choroid, and sclera — rather than within selected subregions. The corresponding tissue-layer areas were measured in Fiji/ImageJ and used for normalization. We have also clarified the limited and exploratory nature of this experiment: only one stained section was available for each location and condition, and the resulting measurements are therefore presented as descriptive exploratory data rather than definitive quantitative comparisons. The revised method section and figure legend now explicitly state the number of animals, sections, and tissue-layer measurement areas analyzed so that the scope of the assessment is transparent.

Revision made in the text**:**

Pages 27-28, lines 814-827, as follow:

"*RNAscope images were acquired using a Nikon Eclipse microscope (Nikon Corporation, Tokyo, Japan) at 20x, 40x, and 100x magnification. Images used for puncta counting were analyzed primarily at 40×, with 100× examination used where necessary to resolve closely clustered signals. Images were opened in Fiji (ImageJ, NIH), and spatial calibration was applied using microscope metadata to set the scale in µm/pixel. For each section, the entire visible area of each tissue layer, retina, choroid, and sclera, was manually outlined using the Freehand Selection tool. These whole-layer outlines were used to define the measurement areas for puncta counting, rather than selecting smaller subregions within each tissue. This procedure was repeated for sections from both the superior and inferior regions of the posterior eye. The outlined tissue-layer areas were measured (µm²) using the Measure function. CYP26A1 RNAscope puncta were counted manually by visual inspection across the entire outlined area of each tissue layer in a blinded manner. For each tissue layer, dot counts were normalized to the corresponding measured tissue-layer area and expressed as puncta per 100,000 µm².*"

2.7.d. The authors should annotate the images to help distinguish retina, choroid, and sclera.

Response:

Yes, this has been done carefully.

2.8. Figure 9 shows western blot analysis with whole protein as the loading control. Despite indicating that the same amount of protein was loaded into each well, it appears that there is significantly more total protein in the PH samples. The authors should address this

Response:

We thank the reviewer for this careful observation. We agree that the total protein image in the representative blot appears stronger in the PH lanes, and we have clarified both the method and quantification approach in the revised manuscript.

First, prior to electrophoresis, protein concentrations for all samples were measured in duplicate by BCA assay, and an equal mass of total protein, 30 µg per lane, was loaded into each basis.

Second, we used post-transfer total protein staining of the membrane for lane-based normalization. This approach was chosen because minor lane-to-lane variation can still arise during electrophoresis, transfer, or membrane staining, even when equal amounts of lysate are loaded. Total protein normalization directly measures the total protein signal present in each lane on the same membrane and is therefore intended to correct for such technical variation. This strategy is also supported by recent work (Westerberg et al. 2025) showing that total protein normalization can provide lower technical variability and a broader reliable dynamic range than several conventional housekeeping proteins, which may vary across samples or biological conditions.

Third, densitometric quantification of CYP26A1 was performed by dividing the target band intensity by the total protein signal from the corresponding lane on the same membrane. Accordingly, if a lane showed a stronger total protein signal, this higher lane signal was incorporated into the denominator during normalization and would reduce, rather than inflate, the normalized CYP26A1 value.

Finally, the bar graph in Figure 9 does not represent raw band intensity from a single blot, but normalized values obtained from three independent biological replicates (n = 3). To avoid confusion, we have revised the Methods section and Figure 9 legend to clarify that total protein staining was performed post-transfer on the membrane and used for lane-based normalization. We have also included the full blot and corresponding total protein images in the Supplementary Materials.

2.9. The authors should address melatonin and its potential influence any comparisons between PH and AH.

Response:

We thank the reviewer for this important point. Melatonin and circadian signaling may influence retinal physiology and ocular growth, and therefore could potentially affect comparisons between PH and AH animals. In the present study, however, all animals were maintained under the same light-dark cycle, and PH and AH tissues were collected at the same clock time within a restricted early-afternoon window (1 pm to 4 pm), in order to minimize circadian variation across groups. We did not directly measure melatonin, melatonin-related signaling, or circadian pathway markers in this study, and therefore cannot exclude a contribution of these mechanisms. We have now added this point in the Discussion.

Revision made:

Page 17, lines 482-486, as follow:

“Although some differences are also likely to reflect pigmentation biology and broader strain-specific background effects, circadian influences, including potential effects of melatonin signaling, cannot be fully excluded in the PH versus AH

comparison, even though this risk was minimized by standardized housing conditions and a restricted tissue-collection window."

Page 24, line 712, as follow:

"All tissue dissections were performed in the early afternoon (1:00–4:00 pm),…"

2.10. In line 564 it appears that there was an editorial comment left in the text "(exactly what pathways)." I agree that these pathways should be described here.

Response:

We thank the reviewer for noting this, we have expanded more explicitly on the principal pathway categories identified in the study.

Revision made:

Page 22, lines 648-649, as follow:

"…, involving extracellular matrix remodeling, focal adhesion, ribosome/translation, metabolic pathways, immune-related signalling, and structural regulation."

2.11. Finally, I do not feel like the title does not match the overall conclusions of the manuscript.

Response:

We thank the reviewer for this helpful comment on the title and for their assessment of the study's overall conclusion. We agree that the original title overemphasized specific interpretive themes that were narrower than the manuscript's broader conclusions. We have therefore revised the title to better reflect the cross-tissue dual-omics framework of the study and its focus on molecular signatures associated with myopia susceptibility and spontaneous myopia.

The revised title would be:

Cross-tissue Dual-omics Analysis Reveals Molecular Programs Linked to Myopia Susceptibility and Progression in a Guinea Pig Model

Reviewer #3 (Comments to the Authors (Required)):

This study by Jiang et al is a valuable approach to better understand the molecular mechanism of myopia. The transcriptional and proteomic analyses are thoroughly performed and provide novel insights into the potential molecular mechanisms of development of myopia. However, the interpretation of the model and some details of the differential expression require further consideration, as outlined below.

We sincerely thank the reviewer for the careful evaluation of our manuscript and for the positive comments on the significance of this study.

We also thank the reviewer for the thoughtful and constructive comments regarding the interpretation of the model and the details of the differential expression analysis. We have addressed these concerns carefully in the revised manuscript. Please see our point-by-point responses below.

Comments:

3.1. The meaning of concordance and discordance, particularly as used in the abstract, should be more explicitly explained (ie RNA and protein expression changing in the same direction).

Response:

We thank the reviewer for this helpful comment. We agree that the terms concordance and discordance should be defined more explicitly in the abstract for clarity. In this study, concordance refers to cases in which transcript and protein expression change in the same direction, whereas discordance refers to cases in which transcript and protein changes are not aligned in direction or are uncoupled. We have revised the abstract accordingly to make this definition clearer for readers.

Revision made:

Page 3, lines 54-55, as follow:

"…*transcript–protein discordance, with RNA and protein changes in remodeling pathways not aligned in direction, …"*

Page 3, lines 57-58, as follow:

", molecular regulation becomes more concordant, with RNA and protein changing in the same direction, indicating…"

3.2. Regulation of gene expression is very complicated, occurring at many levels (transcription, RNA stability, mRNA export, translation efficiency, protein stability etc) Further discussion of this process is required to accompany interpretation of the findings. For example, how does concordance between RNA and protein indicate active remodeling? Discuss the need for a time course experiment to better understand lack of concordance between mRNA and protein levels.

Response:

We agree and have expanded both the Introduction and Discussion to clarify how transcript–protein concordance and discordance should be interpreted biologically. We now explicitly frame discordance as transcriptional priming or post transcriptional buffering, and concordance as indicative of active execution of remodeling programs. We also explain how the dual omics design distinguishes susceptibility from

progression, and acknowledge the limitation of single time point sampling, noting the need for future time course studies.

Revision made:

Introduction, page 5, lines 102 to 116, as follow:

"*To address this limitation, an integrated transcriptomic–proteomic strategy offers the opportunity to examine molecular regulation at complementary levels across multiple ocular tissues. Because gene regulation occurs across multiple levels, including transcription, RNA processing and stability, translational efficiency, and protein turnover, changes in mRNA abundance do not necessarily translate into corresponding protein-level changes (Liu Y et al, 2016). When tissues are collected at a single developmental time point, this layered view may provide indirect insight into regulatory states associated with ongoing ocular growth and remodeling. In this context, transcript–protein discordance may reflect regulatory processes beyond transcription alone, including post-transcriptional buffering (Perl K et al, 2017), or delayed protein accumulation (Fournier ML et al, 2010; Gedeon T & Bokes P, 2012), whereas concordant regulation across both layers is more consistent with coordinated execution of biological programs (Lackner DH et al, 2012). Although time-resolved studies are required to distinguish these possibilities definitively, this framework provides a useful basis for interpreting molecular states associated with susceptibility versus those linked to more active tissue remodeling."*

Discussion, page 18, lines 494–498, as follow:

"*The transition from widespread transcript-protein discordance in the susceptibility state to greater concordance in spontaneous myopia is consistent with a shift from regulatory priming toward more active molecular execution. In this framework, concordance across omics layers is more consistent with engagement of downstream remodeling processes, whereas discordance is more compatible with buffering or preparatory regulatory states."*

Study limitation, page 22, lines 632–633, as follow:

"*Third, transcriptomic and proteomic profiles were assessed at a single developmental time point,..."*

3.3. Evaluation of both protein and RNA expression is a strength of the study. Explain in more detail how this enables distinction of molecular signatures of susceptibility from those of progression. It would be helpful to include an explanation of how discordance between mRNA and protein changes should be interpreted in the Introduction.

Response:

We thank the reviewer for highlighting this strength. We have now explicitly clarified in the Introduction how the dual-omics design enables distinction between molecular signatures associated with susceptibility and those linked to progression. We also specify that susceptibility is characterized by widespread transcript–protein

discordance, consistent with transcriptional priming, whereas progression is marked by increased concordance, consistent with more active molecular execution. These concepts are now described in both the Results overview and the Discussion to improve conceptual clarity.

Revision made:

Introduction, revision was made on lines 102-116, as detailed in our response to Comment 3.2 above.

Results, Overview of analytical framework, page 6, lines 141-146, as follow:

"*This integrative framework enabled susceptibility-associated signatures to be distinguished from progression-associated changes: susceptibility was marked by broad transcriptional alterations not yet consistently reflected at the protein level, consistent with a primed regulatory state, whereas progression showed greater transcript-protein alignment, consistent with more active execution of remodeling programs."*

Discussion, inserted on page 16, lines 430-436, as follow:

"*An important advantage of this approach is that it helps distinguish molecular signatures associated with susceptibility from those linked to progression. In predisposed eyes, widespread transcriptomic changes in choroid and sclera were not yet consistently reflected at the protein level, consistent with a primed regulatory state and possible post-transcriptional buffering. In contrast, spontaneous myopia showed greater transcript-protein concordance, consistent with more active remodeling."*

3.4. Comparison of spontaneously myopic albinos versus their hyperopic littermates is justified to detect differences due to the condition. The comparisons between albino and pigmented animals are described as identifying 'baseline differences linked to susceptibility'. The authors should explain why these differences are linked exclusively to myopia susceptibility rather than presence or absence of pigment? For example, PMEL was unsurprisingly the most consistently differentially expressed between albino and pigmented guinea pigs, but is this gene directly linked to potential development of myopia? I may have misunderstood the design, but pigmented hyperopic were compared with albino hyperopic guinea pigs - if the albinos were hyperopic does this mean that they are the ones that didn't actually spontaneously develop myopia, or are they still at risk of developing myopia if left to live longer?

Response:

We thank the reviewer for this important comment. We agree that the albino hyperopic (AH) vs pigmented hyperopic (PH) guinea pigs' comparison **does not** isolate differences exclusively attributable to myopia susceptibility, and that some signals are likely related to pigmentation or broader strain-specific biology. We have revised the text accordingly to avoid overstatement. Our intention was not to infer

that every DEG identified in AH vs PH is directly involved in myopia development; for example, genes such as PMEL are expected to differ because of pigmentation biology in the choroid. Rather, we use this comparison to identify baseline molecular differences between a more myopia-susceptible albino background and a more myopia-resistant pigmented background. This interpretation is supported by our previous work showing increased susceptibility of albino guinea pigs to deprivation-induced myopia (Figure 2A & B in Jiang et al 2011), as well as by our later study showing that the pigmented strain used here is relatively resistant to myopia-inducing stimuli compared with another pigmented strain (Figure 3 in Jiang et al 2019). We therefore interpret AH as a hyperopic state within a more susceptible background, rather than as a group destined to develop spontaneous myopia in every case.

Revision made:

We have indicated this in Introduction on lines 117-127, as follow:

"*Here, we implemented an integrated multi-omics approach to profile transcriptomic and proteomic signatures across the retina, choroid, and sclera in albino hyperopic (AH), albino spontaneously myopic (AM), and pigmented hyperopic (PH) guinea pigs (GP). Comparison of AH and PH animals, which share a hyperopic state but differ in pigmentation background and susceptibility, was used to identify molecular features associated with myopia predisposition. Comparison of AM and AH littermates was used to identify pathways associated with spontaneous myopia progression. By integrating transcriptomic and proteomic datasets, applying pathway enrichment analyses, and examining patterns of concordance and discordance across ocular tissues from the same eye, this dual-comparison design captures both susceptibility-related and myopia-associated states within the same species.*"

in Results on lines 224-226, as follow:

"…Notably, some overlapping features in the AH vs PH comparison, such as PMEL, are likely to reflect pigmentation-related background differences rather than susceptibility-specific mechanisms."

and in Discussion on lines 480-490, as follow:

"*Together, these findings support a cross-tissue framework in which retinal structural signaling, choroidal neuro-metabolic integration, and scleral immune-metabolic remodeling are associated with myopia susceptibility**, although some observed differences are also likely to reflect pigmentation biology and broader strain-specific background effects……** Subsequent divergence in eye growth, however, is likely to depend not only on these baseline molecular features but also on physiological responses that emerge during the induction phase.*"

3.5. Although the samples clustered by tissue, it is an over-simplification to state that 'all samples within each tissue clustered tightly, with no outliers detected' (Line 129). There is a greater spread within choroid and sclera samples, which although well separated from each other by marker gene

expression, do come close to each other on the PCA plot. Indeed, the spread observed in PC1 is likely due to the extent of contamination with retina tissue (this is on a PCA plot coloured by rhodopsin expression - available on request). Nonetheless, these appear to be high quality samples and this variation does not compromise the analyses, but is probably worth noting. The authors should also assess whether the greater dispersion of choroid and sclera samples might contribute to the discovery of more DEGs than in retina when using low stringency cut-offs for FC and p value.

Response:

We thank the reviewer for this careful and constructive comment. We agree that our original wording overstated the degree of within-tissue clustering. In the revised manuscript, we have replaced this description with a more balanced statement noting that all samples segregated primarily by tissue type, with retina clearly separated from choroid and sclera along PC1, the dominant axis of variation, while the transcriptomic PCA showed greater within-tissue dispersion in choroid and sclera than in retina, particularly along PC2. We now describe this pattern more cautiously as likely reflecting biological heterogeneity together with minor technical and/or cross-layer variation. We also acknowledge the reviewer's suggestion that minor retinal carryover may contribute to this pattern, although the dispersion is observed primarily along PC2 rather than along PC1, which separates retina from the non-retinal tissues.

In response to the reviewer's additional point, we have further noted that the greater dispersion observed in choroid and sclera in the transcriptomic dataset could have contributed, at least in part, to the larger number of DEGs detected in these tissues under the uniform fold-change and p-value thresholds used throughout the study. We retained the same thresholds across all tissues and both comparisons to preserve analytical consistency. Under this framework, DEG yields were already limited in retina and in choroid for the AM vs AH comparison, such that applying substantially stricter thresholds would have risked eliminating biologically informative signals and biasing the analysis toward tissues with stronger signal amplitude. We therefore did not alter the primary DEG definition, but now state explicitly that sample dispersion may have influenced DEG yield across tissues and that this should be considered when interpreting tissue differences in DEG counts. Importantly, tissue-level separation remained clear, marker-based tissue identity supported sample quality, and our main conclusions are based not on DEG counts alone but also on transcript-protein concordance and discordance patterns, cross-tissue comparisons, and pathway-level consistency across datasets.

Revision made:

Page 7, lines 170–180, as follow:

"Although tissue identity was the dominant source of variance, the transcriptomic PCA further indicated greater within-tissue dispersion in choroid and sclera than in retina. All samples segregated primarily by tissue type, with retina clearly separated from choroid and sclera along PC1 (Figure 2A), the dominant axis of variation.

Greater within-tissue dispersion was observed among choroid and sclera samples, particularly along PC2 (Figure 2A), likely reflecting biological heterogeneity together with minor technical and/or cross-layer variation. Importantly, this dispersion did not compromise overall tissue-level separation or downstream analyses. We further note that the greater dispersion in choroid and sclera may have contributed, at least in part, to the larger number of DEGs identified in these tissues under the uniform thresholds applied across comparisons and this should be considered when interpreting differences in DEG counts across tissues."

3.6. More caution required when comparing transcriptomic and proteomic differential expression. The 'striking transcript-protein discrepancy' may be more due in large part to the different sensitivities/biases of the two approaches. More justification required to state that 'large-scale transcriptional priming occurs in the choroid and sclera' (Line 149).

Response:

We thank the reviewer for this important point. We agree that transcriptomic and proteomic datasets differ inherently in sensitivity, coverage, dynamic range, and analytical bias, and that these factors can contribute to incomplete correspondence between differential expression detected at the RNA and protein levels. We have therefore revised the manuscript to use more cautious language and no longer present the observed discordance as evidence of transcriptional priming in a definitive sense. Instead, we now state that the broad transcript-protein discordance observed in choroid and sclera is consistent with a primed or preparatory regulatory state, while acknowledging that technical differences between the two platforms may also contribute to this pattern. At the same time, we do not believe the discordance can be explained solely by methodological differences, because the pattern was tissue- and comparison-dependent rather than uniform across the dataset. In particular, retina showed relatively few DEGs, choroid showed many DEGs in AH vs PH but few in AM vs AH, and sclera remained strongly responsive across both comparisons. Together with clear tissue-level separation and pathway-level structure, these findings support the interpretation that the observed discordance retains biological meaning, while warranting more cautious phrasing.

Revision made:

Pages 7-8, lines 196–202, as follow:

"*This pronounced difference between transcriptomic and proteomic signals may reflect a combination of biological regulation and methodological differences in sensitivity, coverage, and dynamic range between RNA sequencing and mass spectrometry-based proteomics. Consistent with this, the extensive transcriptional changes observed in the choroid and sclera are interpreted here as compatible with a primed regulatory state, with relatively limited translation into stable protein-level differences at this stage."*

3.7. Clarify which parameters were used in which analyses and justify use of

nominal values and small absolute change of 1.2 (20% increase): Transcriptomic analysis. Methods Line 641' Differentially expressed genes were filtered by adjusted p-value and log2 fold-change' Proteomic. Line 662: 'Proteins with llog2 (fold > 0.75 and p-value < 0.05 were considered differentially expressed'. Results Line 143 'Using a threshold of absolute fold-change {greater than or equal to} 1.2 and nominal p {less than or equal to} 0.05, transcriptomic changes were considerably more extensive than proteomic ones.'

Response:

We thank the reviewer for highlighting this lack of clarity. We have now harmonized the cutoff descriptions across the manuscript and clarified that different thresholds were used for distinct analytical purposes, including primary differential expression reporting, descriptive cross-platform comparison, and pathway enrichment. We also now state explicitly that the fold-change threshold of 1.2 in the Results was used for descriptive comparison and visualization rather than for primary statistical inference. The relevant revisions are provided below.

Revision made:

Results, page 7, lines 187 to 191, as follow:

"*For descriptive comparison of transcriptomic and proteomic signal extent across tissues, we used a permissive threshold of absolute fold-change ≥ 1.2 and nominal p ≤ 0.05. Under this criterion, transcriptomic changes were more extensive than proteomic changes. This threshold was used for visualization and cross-platform comparison rather than for primary statistical inference.*"

Methods (Transcriptomics), page 25, lines 748–752, as follow:

"*Differential expression analysis was performed using limma with empirical Bayes moderation. Genes with average counts ≤ 5 were excluded. Differentially expressed genes were identified primarily using adjusted p values for formal reporting, whereas nominal p values and fold changes were used for visualization and rank based downstream analyses.*"

Methods (Proteomics), page 26, lines 774–777, as follow:

"*Given the limited sample size and reduced sensitivity of proteomic measurements relative to RNA sequencing, nominal p values (p < 0.05) combined with a more stringent fold change threshold (|log2FC| > 0.75) were used to identify differentially expressed proteins for integrative analyses.*"

Pathway Analysis, page 27, lines 789–791, as follow:

"*Pathway enrichment analyses were performed using rank-based Gene Set Enrichment Analysis (GSEA), thereby avoiding reliance on arbitrary significance cutoffs and enabling detection of coordinated but modest changes across gene and protein sets.*"

3.8. Provide further justification for the statement that 'these results suggest that while transcriptional regulation shows broad cross-tissue commonalities, proteomic overlap is more restricted' (Line 170). When taking into account the fewer DEPs, is the overlap really significantly less? For example, of 54 DEGs 7 are shared, of 33 DEPs 3 are shared - both close to a proportion of 0.1.

Response:

We thank the reviewer for this important point. We agree that the original wording overstated the difference in cross-tissue overlap between transcriptomic and proteomic datasets. We have therefore revised the text to clarify that the proportional overlap was broadly similar, whereas the absolute number of overlapping transcripts was greater, enabling clearer identification of shared regulatory programs at the transcriptional level. The revised text is provided below.

Revision made:

Results section on page 8, lines 227–231, as follow:

"*Although the proportion of shared features across tissues was broadly similar between transcriptomic and proteomic datasets, the absolute number of overlapping transcripts was greater, facilitating clearer identification of shared regulatory programs at the transcriptional level. By contrast, proteomic overlap was more limited in absolute terms, likely reflecting both lower feature counts and reduced detection sensitivity.*"

3.9. Comment on why 'a larger set of pathways showed strong concordance' line 209.

Response:

We have clarified that pathway-level concordance may reflect aggregation of coordinated gene-level effects that do not necessarily reach significance individually.

More comment has been added in Results on page 10, lines 275–278, as follow:

"*, with pathway-level agreement exceeding gene-level overlap. This likely reflects aggregation of coordinated but individually modest changes across functionally related genes and proteins, making pathway-level convergence a more robust readout of shared regulation.*"

3.10. Comparison with GWAS is a powerful approach. It is striking that all changes in GWAS genes differentially expressed in 'AH vs PH' and 'AM vs AH' change in the opposite direction (Fig 7). For these genes, this suggests that PH is more similar to AM than AH - is this consistent with AH being a susceptible state?

Response:

We thank the reviewer for this insightful comment. We agree that, for the GWAS-overlapping scleral genes in the bulk transcriptomic analysis, the opposite directionality between AH vs PH and AM vs AH indicates that AH should not be viewed as a simple linear intermediate between PH and AM. However, we do not interpret this as inconsistent with AH representing a susceptibility-associated state. Because AH remains phenotypically hyperopic, it may retain protective or compensatory features shared with PH, while also exhibiting molecular alterations that distinguish it from the resistant state. In this framework, susceptibility is not expected to be identical to overt spontaneous myopia. Rather, progression to AM may involve selective reprogramming or loss of protective regulation, rather than simple continuation in the same direction. We therefore interpret AH as a distinct susceptibility-associated state rather than as a uniformly intermediate stage between PH and AM. Although our exploratory RNAscope observations suggest possible local variation not captured by bulk tissue analysis, these image-based data were qualitative and were not used as formal evidence for this interpretation.

This has been discussed on page 21, lines 597 to 604, as follow:

"*In this context, AH should not be viewed as a simple linear intermediate between PH and AM. Rather, AH may represent a distinct susceptibility-associated state that remains phenotypically hyperopic while already showing RA-related regulatory alterations, whereas transition to AM may involve additional reprogramming or loss of compensatory regulation rather than uniform continuation of early changes. Thus, opposite directionality in some GWAS-linked genes does not argue against susceptibility, but instead supports the view that predisposition and progression represent biologically distinct phases.*"

3.11. There are many changes in expression to discuss, but it would be helpful, if possible, to condense this the Discussion section.

Response:

We thank the reviewer for this helpful suggestion. We have condensed the Discussion by reducing detail on individual expression changes and instead emphasizing the main conceptual points, particularly the distinction between susceptibility-associated and progression-associated molecular programs.

3.12. Suggestion for additional work: Look for cell type expression of CYP26A1 in single cell data (mouse?)
Eg consider and cite: Minyu Chen, Jiahui Liu, Guimei Liang, Qingyang Liu, Shuyan Li, Yifang Yang. Cross-species and cross-platform analysis reveals the application value of guinea pig retina in myopia research at single-cell resolution. Experimental Eye Research, Volume 259, 2025, 110558, ISSN 0014-4835. https://ddei5-0-ctp.trendmicro.com:443/wis/clicktime/v1/query?url=https%3a%2f%2fdoi.org%2f10.1016%2fj.exer.2025.110558&umid=832466EF-4BA2-1306-B7C8-960AE62546E4&auth=d4a022de4fa71b6d10c50609f2626ca32dd9d36f-7331a0438ca839e701aac442d6895d73c30befaa

Response:

We thank the reviewer for this helpful suggestion. Cell-type localization of CYP26A1 is clearly of interest, but is beyond the scope of the present bulk-tissue study. We have now acknowledged this as a future direction. We also note that recent single-cell work in guinea pig retina provides a useful retinal resource, although it does not resolve CYP26A1 expression in choroid or sclera, which are central to our current interpretation.

This has been added on page 21, lines 610-615, as follow:

"Integration of bulk multi-omics with single-cell or spatial transcriptomic approaches will be needed to define the cell-type localization of CYP26A1, particularly in choroid and sclera, where the present data indicate transcript-protein discordance. Recent single-cell work in guinea pig retina highlights the feasibility of this direction (Chen M et al, 2025), although equivalent resources for the posterior wall tissues remain limited."

3.13. Minor corrections and suggestions:
3.13.1. Explain what aspect of CYP26A1 is being 'validated' (in Abstract).

Response:

We thank the reviewer for this comment. We agree that the phrase "validation of CYP26A1" was imprecise. Our intention was to indicate validation of the tissue-specific CYP26A1 expression pattern at both the transcript level (RNAscope) and protein level (Western blot), rather than validation of the gene itself. We have revised the Abstract accordingly for clarity.

Revision made:

Page 3, lines 59-62, as follow:
"*Integration with human myopia GWAS loci, **followed by validation of CYP26A1 expression in GP ocular tissues at both transcript and protein levels**, highlights post-transcriptional regulation at the sclera and underscores translational relevance."*

3.13.2. Justify quantification of chemiluminescence signal from western blots. Band densitometry semi quantitative at best.

Response:

We agree with the reviewer that chemiluminescent western blot densitometry is inherently semi-quantitative. Accordingly, our analysis was intended to assess relative differences in CYP26A1 expression between groups rather than to provide absolute quantification. To support the reliability of these comparisons, images were acquired under exposure conditions chosen to avoid obvious saturation, and CYP26A1 band intensity was normalized to the corresponding total protein lane intensity from the same membrane. This approach was used to reduce technical variability related to loading and transfer. The results are therefore presented as

relative fold change across biological replicates, and we have revised the Methods and figure legend to clarify the semi-quantitative nature of this analysis.

Revision made:

Page 28, lines 847-851, as follow:

"*Band densitometry was performed using ImageJ (NIH). CYP26A1 band intensity was normalized to the corresponding total protein signal from the same lane on the same membrane. Total protein was quantified as the integrated density of each sample lane within the detectable molecular weight range, excluding background. Data are presented as relative fold change in normalized CYP26A1 expression from three biological replicates."*

Page 29, lines 860-862, as follow:

"*Western blot results were derived from three independent biological replicates per group and are therefore presented as supportive evidence, to be interpreted with appropriate caution."*

Pages 38-39, lines 1231-1241, as follow:

"Figure 9. Tissue-specific assessment of CYP26A1 expression.

(A) Representative Western blots showing CYP26A1 expression in ocular tissues, with corresponding total protein staining visualized using the No-Stain^{TM} reagent for post-transfer normalization. Full blot images and the corresponding total protein staining are provided in Supplementary Figure 1. (B) ***Densitometric semi quantification*** *of CYP26A1, presented as relative fold change after normalization to the total protein signal of the corresponding lane on the same membrane (mean ± SD). No significant changes were observed in the retina or choroid, whereas scleral CYP26A1 was decreased in both the myopia-prone and spontaneous myopia groups. Data were obtained from a separate cohort of animals (n = 3 per group, 5 weeks old). Statistical significance was assessed by one-way ANOVA followed by Tukey's post-hoc test; *p < 0.05, ***p < 0.001."*

3.13.3. Perhaps an Upset plot might be suitable to illustrate overlap between DEGs/DEPs in the different tissues.

Response:

We thank the reviewer for this helpful suggestion. Because our overlap analyses involved three tissues at a time, we used Venn diagrams as a straightforward visualization of shared and tissue-specific features. We agree that an UpSet plot could also be informative, but we considered the current presentation sufficient for the scale of overlap shown here.

3.13.4. Reword 'In total, 24, 30, and 19 genes were commonly upregulated in the retina-choroid, choroid-sclera, and retina-sclera pairs, respectively' (Line

159). Suggest 'In total, 24 genes were upregulated in retina-choroid, 30 in choroid-sclera, and 19 in retina-sclera comparisons.'

Response:

We thank the reviewer for the suggestion to improve clarity; this has now been revised.

3.13.5. PCA figure - colour in legend to match symbols (red and orange!)

Response:

We thank the reviewer for noting this issue. The symbols were intended to be blue, green, and orange; however, we agree that the orange may appear red in the current version. We have revised the legend to match the symbols.

3.13.6. Include GP = Guinea Pig in abbreviations

Response:

This has been added in line 966.

3.13.7. Line 623: 260/280 nm absorbance ratios (2.11-2.13) on a Nanodrop (Thermo Fisher Scientific) does not measure RNA integrity - it quantifies and assesses purity.

Response:

We thank the reviewer for this correction. We have revised the Methods text accordingly to distinguish these two measures clearly.

Revision made:

Page 25, lines 726-730, as follow:

“RNA purity was assessed by 260/280 nm absorbance ratios (2.11–2.13) on a NanoDrop spectrophotometer (Thermo Fisher Scientific), and RNA integrity was assessed by RNA Integrity Number (RIN = 9.0–10.0) using the RNA Nano 6000 Kit on the Agilent 2100 Bioanalyzer (Agilent, USA).”

3.13.8. Provide more details of default values used for limma, ideally provide script as supplementary. Facilitate replication.

Response:

We have expanded the Methods to specify the limma design, contrasts, and default settings, and provided the full analysis script as a supplementary file.

Revision made:

Methods: more details have been added on page 25, lines 745–747, as follow:

“For limma analyses, normalized log counts were modeled using a linear model with design = ~ batch + condition. Empirical Bayes moderation was applied using default parameters, and contrasts were specified to compare AH vs. PH and AM vs. AH within each tissue.”

and on page 25, lines 752-753, as follow:

“The full limma analysis script is provided as Supplementary File R to facilitate reproducibility.”

3.13.9. Line 414 During the myopia pathogenesis stage, most pathways showed concordant regulation. - clarify that referring to concordance between changes in the three tissues.

Response:

We thank the reviewer for this helpful comment. We have revised the sentence to clarify that, in this context, “concordant regulation” refers to the shared direction of pathway regulation across the retina, choroid and sclera during the spontaneous myopia stage, rather than transcript–protein concordance within a given tissue.

Revision made:

Page 18, lines 498-501, as follow:

“In spontaneously myopic eyes, most pathways were regulated in the same direction across the retina, choroid and sclera, suggesting a more coordinated phase of tissue remodeling associated with myopia.”

May 27, 2026

RE: Life Science Alliance Manuscript #LSA-2025-03595-TR

Dr. Liqin Jiang
Singapore Eye Research Institute
Translational Pre-Clinical Model Platform
The Academia
Singapore 168751
Singapore

Dear Dr. Jiang,

Thank you for submitting your revised manuscript entitled "Cross-tissue dual-omics reveals molecular programs to myopia susceptibility and progression". Your revised manuscript was evaluated by the original reviewers whose comments are appended to this letter. As you will note, the reviewers are in agreement that their previous concerns have been addressed in your revised manuscript. Overall, we would be happy to publish your paper in Life Science Alliance pending final changes necessary to meet our formatting guidelines.

MANUSCRIPT ORGANIZATION AND FORMATTING:

To avoid unnecessary delays in the acceptance and publication of your paper, please read the following information carefully. Full guidelines are available on our Instructions for Authors page, https://www.life-science-alliance.org/authors

Submission of a paper that does not conform to Life Science Alliance guidelines will delay the acceptance of your manuscript.

-Thank you for providing a statement on data availability (line 866). Please remove the sentence on "Data availability" from your abstract (line 64).
-We encourage you to remove the list of abbreviations from the title page and ensure that abbreviations are defined in brackets after their first mention in the text.
-Please add the X and Bluesky handles of your host institute/organization, as well as your own, and/or one of the authors, in our system.
-The titles in both the system and the manuscript file must be consistent with each other.
-In your methods section, please ensure that all relevant studies are cited when making the statement starting line 799, ".....supplemented by GWAS published in PubMed between 2020 and 2022".
-Please make the scale bar and size information for Figure 8E more prominent.
-Please remove sub-headings from the discussion section.
-Please note that the corresponding authors must match between the system and the manuscript file.
-Corresponding authors must connect their accounts with their Orcid IDs.
-Please incorporate any points from the Conclusion section into the Discussion; we only allow a Discussion section.
-Please rename "Disclosure and competing interests statement" to "Conflict of Interest."
-Please add your main, supplementary figure, and table legends to the main manuscript text after the references section.
-Please add an Author Contributions section to your main manuscript text.
-The contributions selected for Audrey W.L. Chia, Seang-Mei Saw, and Veluchamy A. Barathi do not qualify them for authorship. Please either update the contributions in our system and in the Author Contributions section of the manuscript, or let us know if the authors need to be removed (and added eventually to the acknowledgment section)
-Please add callouts for Figures 3A-F; 8A-E and S1A-C to your main manuscript text.
-Please be sure that the authorship listing and order is correct.

We encourage our authors to provide original source data, particularly uncropped/-processed electrophoretic blots and spreadsheets for the main figures of the manuscript. If you would like to add source data, we would welcome one PDF/Excel-file per figure for this information. These files will be linked as supplementary "Source Data" files.

It is Life Science Alliance policy that if requested, original data images must be made available to the editors. Failure to provide original images upon request will result in unavoidable delays in publication. Please ensure that you have access to all original data images prior to final submission.

If you are planning a press release on your work, please inform us immediately to allow informing our production team and scheduling a release date.

We welcome submissions of potential cover images for the issue of LSA in which your work would appear. If you have high quality images associated with this work, please feel free to email these, with a caption, to the journal office.

LSA encourages authors to provide a 30-60 second video where the study is briefly explained. These videos will be appear embedded with the manuscript online at Life Science Alliance, and on social media to promote the published paper and authors (for examples, see https://docs.google.com/document/d/1-UWCfbE4pGcDdcgzcmiuJl2XMBJnxKYeqRvLLrLSo8s/edit?usp=sharing). Corresponding or first-authors are welcome to submit the video. Please submit only one video per manuscript. The video can be emailed to contact@life-science-alliance.org

FINAL FILES:

To upload the final version of your manuscript, please log in to your account: https://lsa.msubmit.net/cgi-bin/main.plex
You will be guided to complete the submission of your revised manuscript and to fill in all necessary information. Please get in touch in case you do not know or remember your login name.

The following items are required for acceptance.

-- An editable version of the final text (.DOC or .DOCX) is needed for copyediting (no PDFs).

-- High-resolution figure, supplementary figure and video files uploaded as individual files: See our detailed guidelines for preparing your production-ready images, https://www.life-science-alliance.org/authors

-- Summary blurb (enter in submission system): A short text summarizing in a single sentence the study (max. 200 characters including spaces). This text is used in conjunction with the titles of papers, hence should be informative and complementary to the title. It should describe the context and significance of the findings for a general readership; it should be written in the present tense and refer to the work in the third person. Author names should not be mentioned.

The license to publish form must be signed before your manuscript can be sent to production. A link to the license to publish form will be available to the corresponding author only. Please take a moment to check your funder requirements.

Reviews, decision letters, and point-by-point responses associated with peer-review at Life Science Alliance will be published, alongside the manuscript. If you do want to opt out of having the reviewer reports and your point-by-point responses displayed, please let us know immediately.

Thank you for your attention to these final processing requirements. Please revise and format the manuscript and upload materials as soon as you are able.

Thank you for this interesting contribution to the literature. We look forward to publishing your paper in Life Science Alliance.

Sincerely,

Sarita Hebbar, PhD
Scientific Editor
Life Science Alliance
http://www.lsajournal.org

Reviewer #1 (Comments to the Authors (Required)):

All my questions have been addressed.

Reviewer #2 (Comments to the Authors (Required)):

The authors have sufficiently addressed my previous concerns.

Reviewer #3 (Comments to the Authors (Required)):

The authors have addressed all the comments, clarifying nomenclature as requested and moderating interpretations such that they are now supported by the data presented.

Dear Dr. Hebbar,

Thank you for your email.

We have made the required corrections. Please find our detailed point-by-point responses below.

Best regards,

-Thank you for providing a statement on data availability (line 866). Please remove the sentence on "Data availability" from your abstract (line 64).

The sentence has been removed.

-We encourage you to remove the list of abbreviations from the title page and ensure that abbreviations are defined in brackets after their first mention in the text.

Thank you for the clarification. The abbreviation list was included at the end of the manuscript rather than on the title page; nevertheless, we have removed the standalone abbreviation list and ensured that abbreviations are defined in brackets at their first mention in the text.

-Please add the X and Bluesky handles of your host institute/organization, as well as your own, and/or one of the authors, in our system.

Thank you for the reminder. We will add the available X and/or Bluesky handles for our host institute/organization and relevant author accounts in the system. For any authors or institutions without available handles, we will leave them blank or indicate that they are not available.

-The titles in both the system and the manuscript file must be consistent with each other.

The title has been corrected and is now consistent between the submission system and the manuscript file.

-In your methods section, please ensure that all relevant studies are cited when making the statement starting line 799, ".....supplemented by GWAS published in PubMed between 2020 and 2022".

Thank you for the reminder. All relevant studies have been cited and updated.

-Please make the scale bar and size information for Figure 8E more prominent.

This has been edited as requested.

-Please remove sub-headings from the discussion section.

All sub-headings have been removed.

-Please note that the corresponding authors must match between the system and the manuscript file.

This has been corrected. The corresponding author information is now consistent between the system and the revised manuscript file.

-Corresponding authors must connect their accounts with their Orchid IDs.

Connections are confirmed.

-Please incorporate any points from the Conclusion section into the Discussion; we only allow a Discussion section.

Thank you for the instruction. We have removed the separate Conclusion section and ensured that relevant concluding points are incorporated within the Discussion. As most of these points were already addressed in the main body of the Discussion, we retained only a brief integrative closing statement at the end of the Discussion:

"Taken together, these findings provide an integrated cross-tissue framework for understanding molecular states associated with myopia susceptibility and spontaneous myopia. They highlight candidate pathways for future mechanistic investigation and support the value of spontaneous myopia as a complementary model for studying biological processes relevant to myopia susceptibility and progression."

-Please rename "Disclosure and competing interests statement" to "Conflict of Interest."

Done.

-Please add your main, supplementary figure, and table legends to the main manuscript text after the references section.

These have been added as instructed.

-Please add an Author Contributions section to your main manuscript text.

Thank you for pointing this out. We have reviewed and updated the Author Contributions section in the main manuscript text and the corresponding contribution selections in the submission system. The revised contributions now more accurately reflect the substantive intellectual, supervisory, resource, analytical, interpretive, and manuscript-review roles of the relevant authors. Based on these updated and confirmed contributions, we would like to retain Audrey W.L. Chia, Seang-Mei Saw, and Veluchamy A. Barathi as co-authors, with Veluchamy A. Barathi remaining as a corresponding author.

-The contributions selected for Audrey W.L. Chia, Seang-Mei Saw, and Veluchamy A. Barathi do not qualify them for authorship. Please either update the contributions in our system and in the Author Contributions section of the

manuscript, or let us know if the authors need to be removed (and added eventually to the acknowledgment section)

Thank you for pointing this out.

We have reviewed the author contribution statements for Audrey W.L. Chia, Seang-Mei Saw, and Veluchamy A. Barathi. Their contributions have now been updated in both the LSA submission system and the Author Contributions section of the manuscript to more accurately reflect their roles in the study.

The revised contribution statements now include their substantive intellectual, supervisory, resource, interpretation, and manuscript review contributions, as applicable. Based on these updated contributions, we confirm that these authors should remain listed as co-authors, and Veluchamy A. Barathi should remain listed as a corresponding author.

Please let us know if any further clarification or adjustment is required.

-Please add callouts for Figures 3A-F; 8A-E and S1A-C to your main manuscript text.

Added.

-Please be sure that the authorship listing and order is correct.

The authorship listing and author order have been carefully checked and confirmed to be correct in both the submission system and the revised manuscript file.

June 10, 2026

RE: Life Science Alliance Manuscript #LSA-2025-03595-TRR

Veluchamy A. Barathi
Singapore Eye Research Institute
Singapore

Dear Dr. Barathi,

Thank you for submitting your Research Article entitled "Cross-tissue dual-omics reveals molecular programs linked to myopia susceptibility and progression". It is a pleasure to let you know that your manuscript is now accepted for publication in Life Science Alliance. Congratulations on this interesting work.

The final published version of your manuscript will be deposited by us to PubMed Central upon publication.

Your manuscript will now progress through copyediting and proofing. As a reminder, the titles in both the system and the manuscript file are not consistent with each other. We will choose the title in the ms file unless you specify otherwise.

It is journal policy that authors provide original data upon request.

Your article will publish open access upon publication under a CC-BY license.

Reviews, decision letters, and point-by-point responses associated with peer-review at Life Science Alliance will be published, alongside the manuscript. If you do want to opt out of having the reviewer reports and your point-by-point responses displayed, please let us know immediately.

IMPORTANT: If you will be unreachable at any time, please provide us with the email address of an alternate author. Failure to respond to routine queries may lead to unavoidable delays in publication.

Scheduling details will be available from our production department. You will receive proofs shortly before the publication date. Only essential corrections can be made at the proof stage so if there are any minor final changes you wish to make to the manuscript, please let the journal office know now.

DISTRIBUTION OF MATERIALS:
Authors are required to distribute freely any materials used in experiments published in Life Science Alliance. Authors are encouraged to deposit materials used in their studies to the appropriate repositories for distribution to researchers.

You can contact the journal office with any questions, contact@life-science-alliance.org

Again, congratulations on a very nice paper. I hope you found the review process to be constructive and are pleased with how the manuscript was handled editorially. We look forward to future exciting submissions from your lab.

Sincerely,

Sarita Hebbar, PhD
Scientific Editor
Life Science Alliance
http://www.lsajournal.org
